# Supplementary material for: Conducting large, repeated, multi-game economic experiments using mobile platforms
Source: PLoS One. 2021 Apr 29;16(4):e0250668. doi: 10.1371/journal.pone.0250668 (PMC8084158; doi:10.1371/journal.pone.0250668)
Supplement: S1 File — Supporting materials contain S1–S22 Tables, S1–S27 Figs, and the experimental instructions. (PDF) [file pone.0250668.s001.pdf]

Supporting Information for Conducting Large, Repeated, Multi-Game Economic Experiments using Mobile Platforms

Zhi Li[1,2], Po-Hsuan Lin[3,4], Si-Yuan Kong[4], Dongwu Wang[4], John Duffy[5,*]

March 21, 2021

[1]Department of Public Finance, School of Economics & the Wang Yanan Institute for Studies in Economics (WISE), Xiamen University, Xiamen, China, 422 Siming S Rd, Siming District, Xiamen, Fujian, China, 361005

[2]The MOE Key Laboratory of Econometrics, Xiamen University, Xiamen, China, 422 Siming S Rd, Siming District, Xiamen, Fujian, China, 361005

[3]Division of the Humanities and Social Sciences, California Institute of Technology, 1200 E California Blvd, Pasadena, CA 91125, USA

[4]MobLab Inc., 380 N Halstead St, Pasadena, CA 91107, USA

[5]Department of Economics, University of California, Irvine, 3151 Social Sciences Plaza, Irvine, CA 92697-5100, USA

[*]Correspondence author: John Duffy (`duffy@uci.edu`)

Supplementary Information

A Data 1

B Supplementary Individual Game Analysis 5

B.1 Beauty Contest Game . . . 5
B.2 Voter Turnout Game . . . 9
B.3 Public Goods Game . . . 11
B.4 Ultimatum Game . . . 17
B.5 Risk Elicitation . . . 20
B.6 Centipede Game . . . 22
B.7 Trust Game . . . 24

C Gender 26

C.1 Beauty Contest Game . . . 26
C.2 Ultimatum Game . . . 27
C.3 Risk Elicitation Task . . . 28
C.4 Centipede Game . . . 30
C.5 Trust Game . . . 32
C.6 Math Competition . . . 34

D Behavior Across Games 36

D.1 Subsample Analysis . . . 36
D.2 Spearman's Rank-Order Correlation Coefficients . . . 39

E Method 41

E.1 Background of Participants and Experimental Implementation . . . 41
E.2 Instruction Slides . . . 43
E.3 Game Configurations . . . 49

Supplementary Tables 1-22
Supplementary Figures 1-27

A Data

S1 Table: Number of Participants in Each Game

	Experiment 1 Large	Experiment 1 Small	Experiment 2 Large	Experiment 2 Small
Total # of Participants	393	240	402	183
Games[1]				
Beauty Contest[2]	198	137	378	169
	(2)	(15)	(4)	(17)
Voter Turnout Game[3]	388	224	383	170
	(4)	(23)	(4)	(17)
Public Good Game[4]	388	235	390	170
	(4)	(24)	(4)	(17)
Ultimatum Game	384	231	383	170
Risk Elicitation	378	229	386	173
Centipede Game	363	217	365	164
Trust Game	364	226	379	172
Math Competition	386	234	380	171

[1] This table shows the number of players included in the analysis. For the beauty contest game, the voter turnout game and the public good game, the number of groups are shown in the parenthesis.

[2] For the beauty contest game, we exclude two large groups (with only 22 and 48 players) and one small group (with only 3 players) in Experiment 1. In Experiment 2, one large group (with only 1 player) and one small group (with only 5 players) are excluded from the analysis.

[3] For the voter turnout game, one small group with only 3 players in Experiment 2 is excluded from the analysis.

[4] For the public good game, one small group (with only 3 players) in Experiment 1 and one small group (with only 4 players) are excluded from the analysis.

This data set consists of the survey data and game-play data from Experiment 1 and Experiment 2. In this study, we use and analyze data from players who participated in at least one of the eight games. Since the whole experiment is implemented on players' mobile phones, it is possible that the players leave the app during the experiment. In S1 Table, we report the number of participants in each game of our analysis.

Moreover, from the pre-game survey, we are able to collect demographic data on each player's gender, place of origin, cognitive reflection test (CRT) score and their scores on China's National College Entrance Examination (NCEE), commonly known as the Gaokao score. In

S2 Table, we report the summary statistics of thse demographic variables. In addition, we summarize the players' provinces of birthplace and school in S3 Table . Finally, in S4 Table, we describe the key variables in the analysis.

S2 Table: Summary Statistics of Demographic Variables

	N	Missing	Mean	SD	Median	Max	min
Experiment 1							
Female	631	2	0.743	0.437	1	1	0
CRT Score	629	4	0.876	0.225	1	1	0
Gaokao Score	532	101	590.2	70.95	607.0	762	360
Experiment 2							
Female	566	19	0.781	0.414	1	1	0
CRT Score	579	6	0.877	0.235	1	1	0
Gaokao Score	564	21	583.0	70.55	599.5	1000	349

S3 Table: Number of Observations by Birthplace and School

	Birthplace		School	
Province	Experiment 1	Experiment 2	Experiment 1	Experiment 2
Anhui	40	21	35	23
Beijing	1	22	10	39
Chongqing	14	9	17	7
Fujian	79	128	71	90
Gansu	9	3	4	1
Guangdong	14	16	17	18
Guangxi	12	6	8	3
Guizhou	5	2	5	2
Hainan	2	3	3	4
Hebei	23	6	31	6
Heilongjiang	5	6	7	0
Henan	40	14	34	8
Hubei	29	83	12	51
Hunan	43	65	34	53
Inner Mongolia	7	1	5	1
Jiangsu	46	32	40	34
Jiangxi	39	13	31	13
Jilin	8	23	9	22
Liaoning	14	25	18	14
Ningxia	1	2	1	1
Qinghai	1	1	0	1
Shaanxi	12	19	6	19
Shandong	52	8	29	13
Shanghai	4	7	4	11
Shanxi	24	0	23	0
Sichuan	49	103	59	129
Tianjin	1	8	5	6
Xinjiang	3	2	1	1
Yunnan	8	1	9	1
Zhejiang	44	2	38	1
Missing	4	2	19	13

S4 Table: Summary of Variables

Variables	Description
Basic Variables	
Female	Dummy variable equals to 1 if the player is female.
Gaokao Score	Self-reported Gaokao score standardized around the sample average.
CRT Score	The correction rate of cognitive reflection test.
Risk Aversion	# of safe options chosen in risk elicitation task standardized around the sample average.
Gameplay Math Score	The higher number of correct puzzles in the first two stages of the math competition game standardized around the sample average.
Tournament	Dummy variable equals to 1 if the player chooses "tournament" in the math competition game.
Strategic Behavior	
Initial Guess	The first guess in the beauty contest game.
Follow BNE	$\frac{1}{N_i}\sum_{j=1}^{N_i}\mathbb{1}\{i$ follows EQ cutoff strategy in round $j\}$ where N_i is the number of rounds that player i has participated.
Node 1 Take Rate	The average take rate at the first node of the centipede game.
Node 2 Take Rate	The average take rate at the second node of the centipede game (conditional on player 1 passes).
Strong Free Rider	$\frac{1}{N_i}\sum_{j=1}^{N_i}\mathbb{1}\{\text{contribution in round } j = 0\}$ where N_i is the number of rounds that player i has participated.
Free Rider	$\frac{1}{N_i}\sum_{j=1}^{N_i}\mathbb{1}\{\text{contribution in round } j \leq 6\}$ where N_i is the number of rounds that player i has participated.
Altruist	$\frac{1}{N_i}\sum_{j=1}^{N_i}\mathbb{1}\{\text{contribution in round } j \geq 14\}$ where N_i is the number of rounds that player i has participated.
Proposal Offer	Proportion of pie offered to the responder in the ultimatum game.
Acceptance	Dummy variable equals to 1 if the responder accepts the offer.
Investment	Proportion of endowment offered to the second mover in the trust game.
Return	The ratio between the amount of return and the amount of investment from the investor in the trust game (conditional on that the investor does not invest 0).

B Supplementary Individual Game Analysis

B.1 Beauty Contest Game

S5 Table: Beauty Contest Summary Statistics

	Experiment 1				Experiment 2			
	N	Mean	SD	Median	N	Mean	SD	Median
Group A (Large)								
Round 1	178	31.58	20.23	30.0	357	35.80	20.42	34.0
Round 2	188	24.14	15.75	20.0	372	25.27	15.96	22.0
Round 3	190	14.31	9.366	12.0	374	16.18	12.03	13.0
Group B (Small)								
Round 1	115	37.39	22.05	34.0	166	38.75	22.14	35.5
Round 2	124	32.25	19.39	28.5	162	29.59	18.18	25.0
Round 3	122	24.31	14.83	21.0	162	20.97	15.59	18.0

1. In Experiment 1, there are 4 groups in Group A and 16 groups in Group B. Due to a connection error, there are 2 groups in Group A that have less than 70 players and 1 group in Group B having less than 7 players. These 3 groups are excluded from the group size effect analysis.
2. In Experiment 2, there are 5 groups in Group A and 18 groups in Group B. There is one group in Group A having only one player and and one group in Group B having only five players due to the lagged connection. These two groups are excluded from the group size effect analysis.

S6 Table: P-values of Kolmogorov-Smirnov Tests

Large vs. Small		Exp 1 vs. Exp 2	
Experiment 1		*Large Group*	
Round 1	0.080	Round 1	0.004
Round 2	0.000	Round 2	0.407
Round 3	0.000	Round 3	0.074
Experiment 2		*Small Group*	
Round 1	0.251	Round 1	0.669
Round 2	0.091	Round 2	0.199
Round 3	0.000	Round 3	0.009

In this section, we provide further evidence in favor of the differential effect of group size on convergence to the equilibrium prediction. First of all, in S5 Table, we report the summary statistics of the guesses and the p-values of Kolmogorov-Smirnov tests are summarized in S6 Table. From the table, we can observe that the group size effect is more significant in the later rounds and guesses are similar in Experiments 1 and 2. S1 and S2 Figs show the scatter plots of the second guesses against the first guesses and scatter plots of the third guesses against the second guesses for both the Large groups (first column) and Small groups (second column) in Experiments 1 and 2. Moreover, we conduct the same analysis on the data from Nagel (1995) (third column) in order to compare our result with the literature.

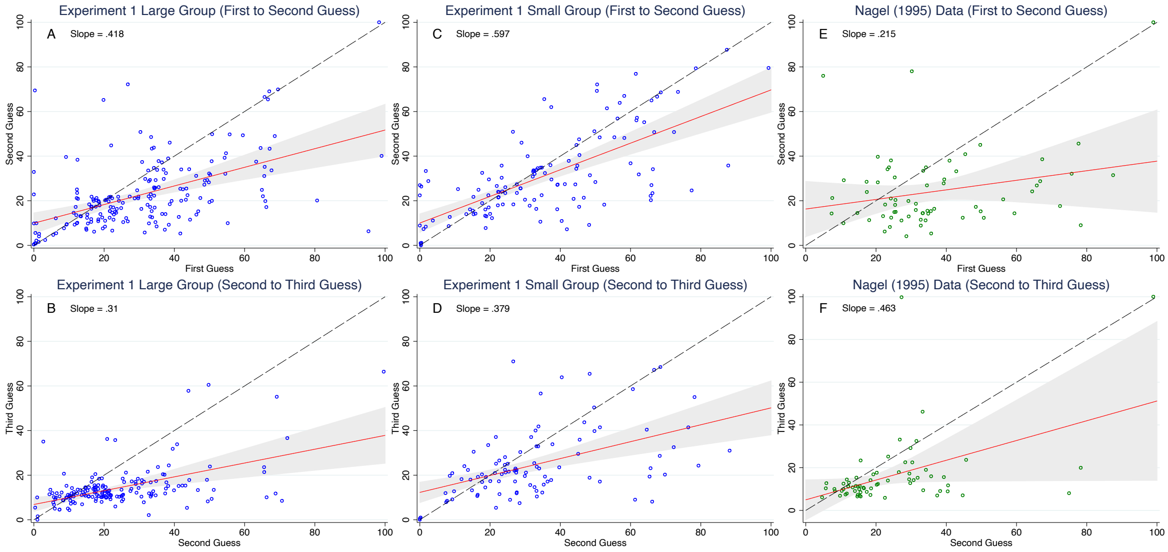

S1 Fig: Observations over time from Experiment 1. Figure A and C show the transitions from the first guess to the second guess. Figure B and D two figures show the transitions from the second guess to the third guess. Figure E and F show the transitions in Nagel (1995). The black dotted line is the 45 degree line and the red line is the best fitted line overlaid with 95% CI.

The figures clearly reveal that guesses are more closely clustered in the neighborhood of zero in both transition graphs of the Large groups as compared with the Small groups indicating a faster convergence in the latter. Moreover, we can actually quantify the speed of convergence from the change of slopes. In the Large groups of Experiment 1, the slopes are 0.418 ($\hat{\beta} = 0.418, t = 5.27, p < 0.001, 95\%$ C.I. $= [0.261, 0.574]$) and 0.310 ($\hat{\beta} = 0.310, t = 3.97, p < 0.001, 95\%$ C.I. $= [0.156, 0.464]$) which are smaller than the slopes for the Small

groups which are 0.597 ($\hat{\beta} = 0.597, t = 8.91, p < 0.001, 95\%$ C.I. $= [0.464, 0.730]$) and 0.379 ($\hat{\beta} = 0.379, t = 4.77, p < 0.001, 95\%$ C.I. $= [0.221, 0.537]$), indicating the convergence in Large groups is faster than the Small groups.

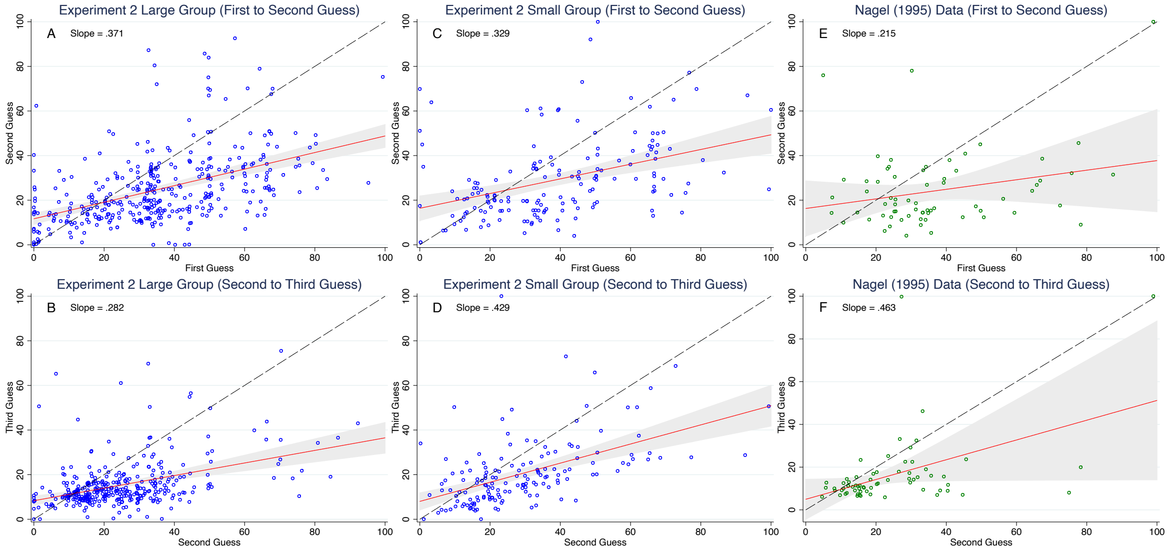

S2 Fig: Observations over time from Experiment 2. Figure A and C show the transitions from the first guess to the second guess. Figure B and D two figures show the transitions from the second guess to the third guess. Figure E and F show the transitions in Nagel (1995). The black dotted line is the 45 degree line and the red line is the best fitted line overlaid with 95% CI.

In addition, we can find that the speed of convergence in Experiment 2 is quantitatively similar to Experiment 1. The slopes of the Large groups are 0.371 ($\hat{\beta} = 0.371, t = 10.39, p < 0.001, 95\%$ C.I. $= [0.301, 0.441]$) and 0.282 ($\hat{\beta} = 0.282, t = 6.43, p < 0.001, 95\%$ C.I. $= [0.196, 0.368]$). On the other hand, the slopes of the Small groups are 0.329 ($\hat{\beta} = 0.329, t = 5.10, p < 0.001, 95\%$ C.I. $= [0.202, 0.457]$) and 0.429 ($\hat{\beta} = 0.429, t = 6.98, p < 0.001, 95\%$ C.I. $= [0.308, 0.550]$). This shows that in Experiment 2 the convergence in Large groups is also faster than the Small groups.

Finally, compared with the data from Nagel (1995), we can observe that the speed of convergence is quantitatively similar in both experiments. While the convergence from the first round to the second round in Nagel (1995) is smaller ($\hat{\beta} = 0.215, t = 1.25, p = 0.216, 95\%$ C.I. $= [-0.129, 0.558]$), the convergence from the second round to the third round is indeed comparable ($\hat{\beta} = 0.463, t = 2.01, p = 0.048, 95\%$ C.I. $= [0.003, 0.922]$). This comparison demonstrates the robustness of our result in terms of the speed of convergence.

S3 Fig reports on our classification of players by strategic sophistication using the initial guesses in the beauty contest game. Here we follow Nagel (1995) to assign level 0 status to those whose guess is greater than the midpoint of the interval, i.e. L0 = [50, 100], and then proceeds iteratively using the midpoint of 50, so that L1 types are those whose guesses are less than 50 but greater than or equal to $2/3 \times 50$, or in the range [33.33, 50); L2 players have guesses in [22.22, 33.33); L3 players have guesses in [14.8, 22.1] and >L3 types have guesses in [0, 14.7]. S3 Fig shows the distribution of both level-k type classifications in both Experiments 1 and 2. The result shows that level 0 is the most common type, followed by levels 1 then 2 then 3, which is a common finding in this literature, e.g. as in Nagel (1995). In addition, the distributions in Experiments 1 and 2 are not significantly different (Kolmogorov-Smirnov Test: $KS = 0.0727$, $p = 0.148$).

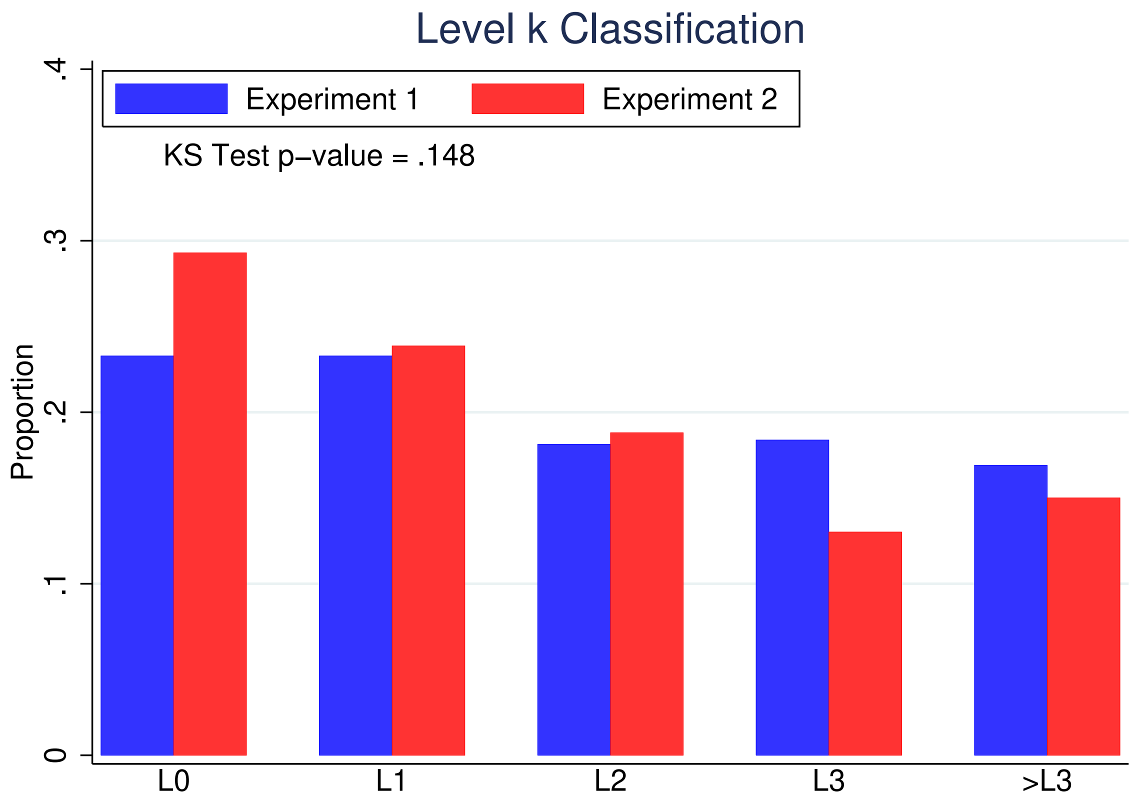

S3 Fig: The distribution of levels based on the initial guess in the beauty contest game. The blue bars and red bars show the classification results from Experiments 1 and 2, respectively. The p-value of the Kolmogorov-Smirnov test is provided in the figure.

B.2 Voter Turnout Game

In this section, we report the summary statistics for the voter turnout game in S7 Table, which are visualized in Figure 2 of the main text. Moreover, to examine the group size effect and the consistency between Experiment 1 and 2, we conduct Mann-Whitney ranksum tests and report the p-values in S8 Table. From the table, we can observe that the results are similar in Experiments 1 and 2 but we can only find weak group size effects.

S7 Table: Voter Turnout Game Summary Statistics

	Experiment 1 Large			Experiment 1 Small			Experiment 2 Large			Experiment 2 Small		
	N	Mean	SD	N	Mean	SD	N	Mean	SD	N	Mean	SD
Majority												
Round 1	215	0.698	0.460	141	0.780	0.416	246	0.764	0.425	116	0.828	0.379
Round 2	244	0.672	0.470	154	0.701	0.459	248	0.677	0.468	114	0.702	0.460
Round 3	252	0.552	0.498	147	0.667	0.473	251	0.518	0.501	116	0.586	0.495
BNE Prediction		0.101			0.258			0.101			0.258	
Minority												
Round 1	109	0.615	0.489	57	0.649	0.481	123	0.431	0.497	51	0.608	0.493
Round 2	120	0.483	0.502	66	0.591	0.495	126	0.429	0.497	49	0.347	0.481
Round 3	124	0.403	0.493	63	0.492	0.504	124	0.371	0.485	50	0.340	0.479
BNE Prediction		0.123			0.292			0.123			0.292	

[1.] There is one small group in Experiment 2 being excluded from the analysis since the group has only three players.

S8 Table: P-values of Mann–Whitney Ranksum Tests

	Large vs. Small		Exp 1 vs. Exp 2
Experiment 1 (Majority)		*Large Group (Majority)*	
Round 1	0.0868	Round 1	0.1074
Round 2	0.5428	Round 2	0.9005
Round 3	0.0241	Round 3	0.4497
Experiment 1 (Minority)		*Large Group (Minority)*	
Round 1	0.6641	Round 1	0.0053
Round 2	0.1611	Round 2	0.3896
Round 3	0.2478	Round 3	0.6028
Experiment 2 (Majority)		*Small Group (Majority)*	
Round 1	0.1718	Round 1	0.3436
Round 2	0.6438	Round 2	0.9936
Round 3	0.2230	Round 3	0.1802
Experiment 2 (Minority)		*Small Group (Minority)*	
Round 1	0.0340	Round 1	0.6589
Round 2	0.3248	Round 2	0.0100
Round 3	0.7013	Round 3	0.1059

B.3 Public Goods Game

In this section, we report the summary statistics for the public goods game in Supplementary Tables 9 and 10, which are visualized in Figure 3 of the main text. The summary statistics for the amount of contributions and the proportion of altruists are reported in S9 Table. Besides from analyzing the free-riding behavior, we further separate out the "strong free-riding behavior"—which is the frequency that a player contributes exactly 0 to the public good. The summary statistics of (strong) free-riding behavior are reported in S10 Table

Moreover, in order to test the existence of the group size effect, we conduct Mann-Whitney ranksum tests (where large and small groups having the same median is the null hypothesis) and report the p-values in S11 Table. Finally, in S12 Table, we compare the results in Experiment 1 and Experiment 2 by reporting the p-values of Mann-Whitney ranksum tests.

The result shows that the group size only has mild effect on the experimental results and this is finding is replicated in Experiment 2. In order to understand why group size does not seem to matter in our experiment, in S4 Fig we also report on the percentage of certain player types each round and overall rounds that we could identify using our data. Following Isaac et al. (1994), players are classified as strong free riders if they give 0 in a round; a (weak) free rider if they give less than 1/3 of their endowment in a round and an altruist if they give at least 2/3 of their endowment in a round. The overall round classification (S4 Fig (1E) and (2E)) requires that a player was a certain type for at least 1/2 of all rounds of the game.

While we generally observe little to no difference in the percentages of (weak) free riders between the Large and Small group treatments, we find that in the Large group treatment we have more Strong free riders and Altruists than in the Small group treatment (Experiment 1 χ^2-test: $\chi^2(3) = 16.091$, $p = 0.001$; Experiment 2 χ^2-test: $\chi^2(3) = 6.928$, $p = 0.074$). That is, there is a greater heterogeneity of player types in the Large group treatment; the greater numbers of Altruists and Strong Free riders in the Large group treatment off-set one another so that on average, there is no difference in group contributions between the Large and Small groups. Compared with the data from Isaac et al. (1994), we can also observe the same pattern in their data—when the group size gets larger, the more heterogeneous the player types are.

S9 Table: Average proportion of endowment contributed and alutrists

	Experiment 1 Large			Experiment 1 Small			Experiment 2 Large			Experiment 2 Small		
	N	Mean	SD	N	Mean	SD	N	Mean	SD	N	Mean	SD
% of Endowment Contributed												
Round 1	356	0.345	0.279	224	0.314	0.246	311	0.254	0.242	151	0.311	0.253
Round 2	369	0.382	0.297	226	0.350	0.258	369	0.285	0.275	162	0.282	0.243
Round 3	362	0.381	0.333	222	0.355	0.264	375	0.312	0.314	160	0.269	0.256
Round 4	359	0.361	0.337	220	0.341	0.267	377	0.319	0.328	164	0.274	0.266
Round 5	358	0.305	0.327	220	0.307	0.249	379	0.298	0.322	164	0.256	0.271
Round 6	360	0.252	0.308	223	0.261	0.240	379	0.249	0.290	167	0.222	0.262
Round 7	369	0.253	0.316	226	0.221	0.242	377	0.214	0.285	168	0.208	0.239
Round 8	245	0.210	0.305	214	0.197	0.251	379	0.183	0.277	170	0.181	0.229
% of Altruists												
Round 1	356	0.118	0.323	224	0.103	0.304	311	0.058	0.234	151	0.079	0.271
Round 2	369	0.171	0.377	226	0.111	0.314	369	0.092	0.290	162	0.068	0.252
Round 3	362	0.213	0.410	222	0.144	0.352	375	0.163	0.370	160	0.069	0.254
Round 4	359	0.209	0.407	220	0.114	0.318	377	0.170	0.376	164	0.085	0.280
Round 5	358	0.156	0.364	220	0.077	0.268	379	0.161	0.368	164	0.085	0.280
Round 6	360	0.119	0.325	223	0.076	0.266	379	0.103	0.304	167	0.084	0.278
Round 7	369	0.127	0.334	226	0.066	0.249	377	0.101	0.301	168	0.042	0.200
Round 8	245	0.127	0.333	214	0.075	0.264	379	0.082	0.274	170	0.041	0.199

[1] Notice that Altruist is a dummy variable which equals to 1 when the proportion of endowment contributed is greater or equal to 2/3.

[2] We exclude one small group in Experiment 1 (with only 3 players) and one small group in Experiment 2 (with only 4 players) from the analysis.

S10 Table: Average proportion of (strong) free riders

	Experiment 1 Large			Experiment 1 Small			Experiment 2 Large			Experiment 2 Small		
	N	Mean	SD	N	Mean	SD	N	Mean	SD	N	Mean	SD
% of Strong Free Rider												
Round 1	356	0.124	0.330	224	0.107	0.310	311	0.215	0.412	151	0.166	0.373
Round 2	369	0.133	0.340	226	0.097	0.297	369	0.209	0.407	162	0.173	0.379
Round 3	362	0.193	0.395	222	0.117	0.322	375	0.243	0.429	160	0.238	0.427
Round 4	359	0.242	0.429	220	0.127	0.334	377	0.263	0.441	164	0.213	0.411
Round 5	358	0.302	0.460	220	0.168	0.375	379	0.266	0.443	164	0.238	0.427
Round 6	360	0.333	0.472	223	0.202	0.402	379	0.322	0.468	167	0.293	0.457
Round 7	369	0.333	0.472	226	0.235	0.425	377	0.363	0.482	168	0.292	0.456
Round 8	245	0.412	0.493	214	0.327	0.470	379	0.417	0.494	170	0.341	0.476
% of Free Rider												
Round 1	356	0.559	0.497	224	0.634	0.483	311	0.684	0.465	151	0.589	0.494
Round 2	369	0.491	0.501	226	0.535	0.500	369	0.631	0.483	162	0.660	0.475
Round 3	362	0.506	0.501	222	0.527	0.500	375	0.624	0.485	160	0.675	0.470
Round 4	359	0.535	0.499	220	0.550	0.499	377	0.605	0.490	164	0.677	0.469
Round 5	358	0.623	0.485	220	0.595	0.492	379	0.633	0.483	164	0.695	0.462
Round 6	360	0.714	0.453	223	0.655	0.477	379	0.691	0.463	167	0.766	0.424
Round 7	369	0.732	0.444	226	0.765	0.425	377	0.748	0.435	168	0.774	0.420
Round 8	245	0.784	0.413	214	0.776	0.418	379	0.807	0.395	170	0.806	0.397

1. Strong free rider is a dummy variable which equals to 1 when the player exactly contributes 0.

2. Free rider is a dummy variable which equals to 1 when the proportion of endowment contributed is less or equal to 1/3.

3 We exclude one small group in Experiment 1 (with only 3 players) and one small group in Experiment 2 (with only 4 players) from the analysis.

S11 Table: P-values of Mann–Whitney Ranksum Tests

	Large vs. Small			
	Contribution	Altruist	Strong Free Rider	Free Rider
Experiment 1				
Round 1	0.3237	0.5699	0.5491	0.0745
Round 2	0.2994	0.0452	0.1959	0.2883
Round 3	0.9516	0.0392	0.0159	0.6141
Round 4	0.7157	0.0033	0.0008	0.7222
Round 5	0.0882	0.0055	0.0003	0.5111
Round 6	0.0187	0.0954	0.0006	0.1331
Round 7	0.4783	0.0182	0.0104	0.3596
Round 8	0.2922	0.0683	0.0601	0.8372
Experiment 2				
Round 1	0.0144	0.3775	0.2085	0.0433
Round 2	0.6520	0.3562	0.3403	0.5210
Round 3	0.4750	0.0036	0.8983	0.2612
Round 4	0.5564	0.0103	0.2236	0.1116
Round 5	0.5849	0.0192	0.4833	0.1650
Round 6	0.6379	0.4884	0.5088	0.0735
Round 7	0.2548	0.0207	0.1032	0.5177
Round 8	0.1446	0.0833	0.0935	0.9671

S12 Table: P-values of Mann–Whitney Ranksum Tests

	Experiment 1 vs. Experiment 2			
	Contribution	Altruist	Strong Free Rider	Free Rider
Large Groups				
Round 1	0.0000	0.0068	0.0015	0.0009
Round 2	0.0000	0.0016	0.0062	0.0001
Round 3	0.0035	0.0819	0.1056	0.0012
Round 4	0.0867	0.1752	0.5275	0.0555
Round 5	0.9460	0.8667	0.2899	0.7717
Round 6	0.7444	0.4745	0.7408	0.5022
Round 7	0.1252	0.2537	0.3893	0.6120
Round 8	0.4746	0.0683	0.9086	0.4715
Small Groups				
Round 1	0.8387	0.4492	0.1002	0.3852
Round 2	0.0101	0.1532	0.0288	0.0137
Round 3	0.0010	0.0216	0.0019	0.0038
Round 4	0.0048	0.3649	0.0243	0.0121
Round 5	0.0067	0.7737	0.0907	0.0447
Round 6	0.0166	0.7840	0.0366	0.0170
Round 7	0.4032	0.2915	0.2008	0.8465
Round 8	0.7169	0.1688	0.7717	0.4721

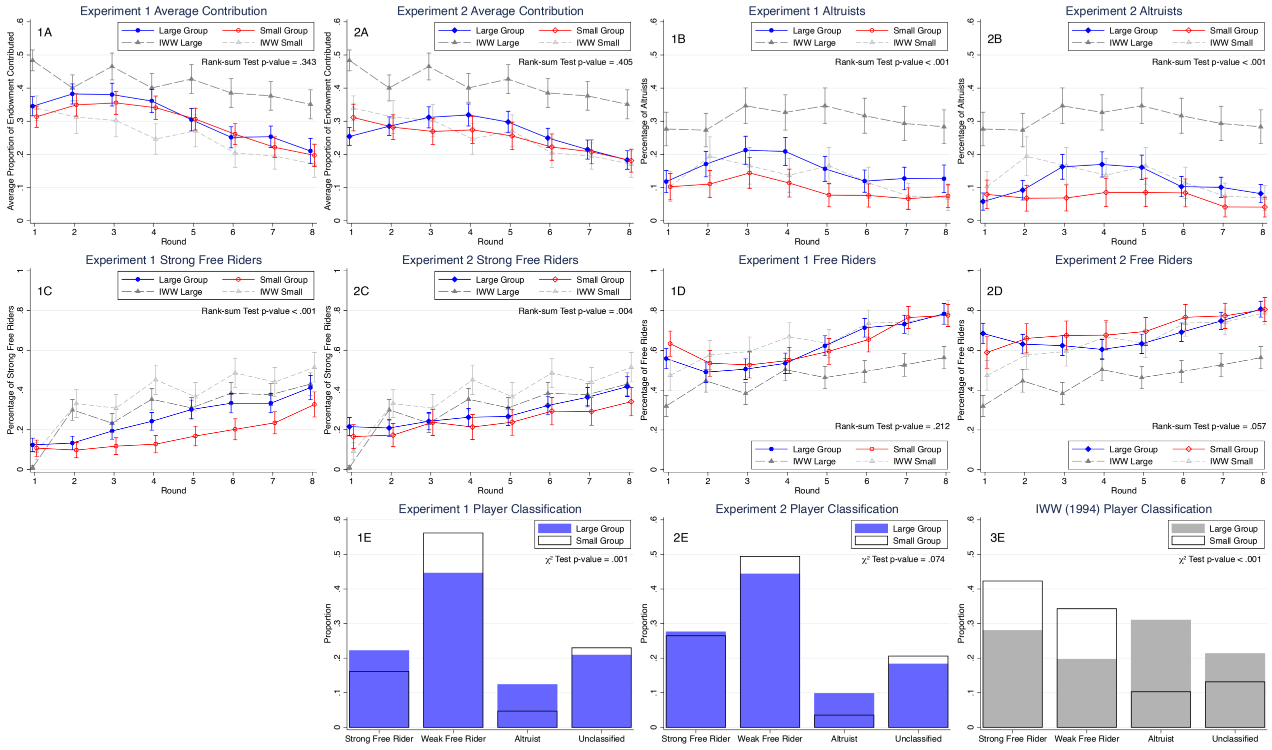

S4 Fig: Average contribution, proportion of (strong) free-riding and altruists and player classifications in the public good games. (1A-1D) The average proportion of endowment contributed, proportion of (strong) free-riding and altruists (overlaid with 95% CI) across different rounds in Experiment 1 with the p-values from a Mann-Whitney test on pooled data over all eight rounds. (2A-2D) The average proportion of endowment contributed, proportion of (strong) free-riding and altruists (overlaid with 95% CI) across different rounds in Experiment 2 with the p-values from a Mann-Whitney test on pooled data over all eight rounds. Moreover, in these 8 panels, we overlay the data from Isaac et al. (1994) with gray lines. (1E, 2E and 3E) The classification results of Experiments 1, 2 and the experimental data from Isaac et al. (1994) with the p-values of χ^2 tests.

B.4 Ultimatum Game

S13 Table: Ultimatum Game Summary Statistics

	N	Mean	SD	Median
Experiment 1				
Proposal Offer	313	36.22	15.47	40.0
Acceptance	302	0.699	0.460	1
Experiment 2				
Proposal Offer	277	36.91	15.68	40.0
Acceptance	276	0.775	0.418	1

The fourth game in our playlist was a two-player ultimatum game, originally studied by Güth et al. (1982) and subsequently by many others. In this 2-player, take-it-or-leave-it bargaining game, the first mover proposes a split of a pie of 100 points between herself and the second mover. The second mover sees the proposed split and must decide whether to accept the proposal or reject it. In the case of acceptance, the pie is split between the first mover and the second mover in accordance with the first mover's proposed allocation. If the second mover rejects, then both players earn 0. As this is an one-shot game, working backwards, the second mover should accept any positive offer by the first mover and is indifferent between a 0 offer and rejection. Thus, the strict subgame perfect Nash equilibrium calls for the first mover to make the minimum positive offer (here 1 point) and for the second mover to accept this offer. We had all subjects in our experiment play this 2-player game just one time, with random assignment to either the first or the second mover roles. The results are shown in S5 Fig.

The first two panels show the distributions of proposal offers from the first mover offered to the second mover and the second mover's acceptance rate, conditional on the offered amount. Notice that offers to the second mover are nearly always 50 percent or less of the pie size ($\leq$ 50 points), and the conditional acceptance rate is monotonically increasing with the amount offered to the second mover. This finding is consistent with the literature, e.g. Roth et al. (1991) and Lin et al. (2020). Moreover, in the bottom two panels, we compare our data with Roth et al. (1991) by conducting the same analysis. From the figure, we can observe that no matter in the distribution of proposal offers or the conditional acceptance rate, both data sets obtain similar results, showing the robustness of our result.

S6 Fig[1] shows the second mover (responder)'s *reaction times* (in sec-

[1]To ensure our analysis is not skewed by extreme values, we drop 7 observations in Experiment 1 that take

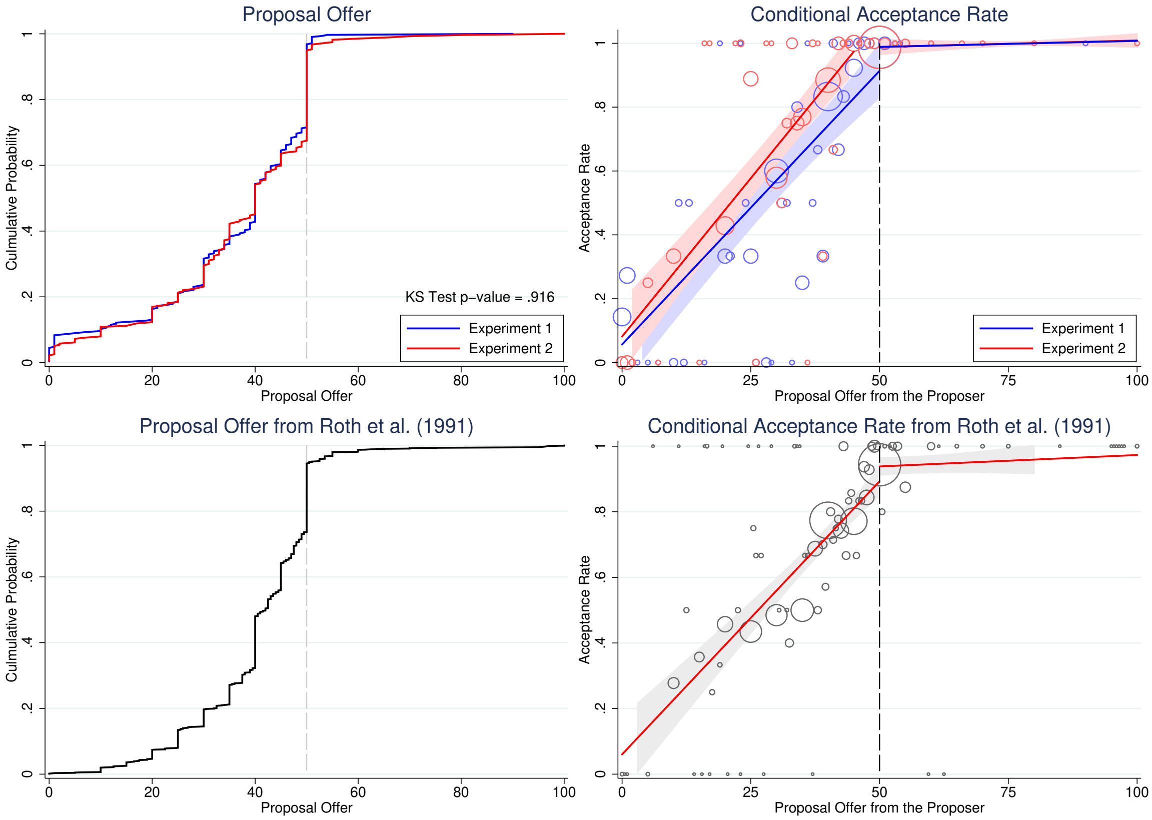

S5 Fig: Proposal offer and conditional acceptance rates. The blue curves and red curves show the distribution of proposal offers and conditional acceptance rates from Experiment 1 and 2, respectively. The bottom two panels show the distribution of proposal
offers from Roth et al. (1991).

onds) before either accepting or rejecting a proposal, again conditional on the amount of that proposal. No matter in Experiment 1 or 2, we observe that acceptance reaction times have a huge drop when approaching the equal-split allocation, indicating the special appeal of the equal-split allocation (see Lin et al. (2020)).

Moreover, in Experiment 1, the trend of the reaction time (conditional on accepting) shows an inverted U-shape peaking at around 30% which they are indifferent between accepting and rejecting. This observation is consistent with Chabris et al. (2009), Konovalov and Krajbich (2019) and Krajbich et al. (2014).

more than 88 seconds (= Q3 + 3 × IQR) to respond. Similarly, in Experiment 2, we drop 6 observations that take more than 90 seconds (= Q3 + 3 × IQR) to respond.

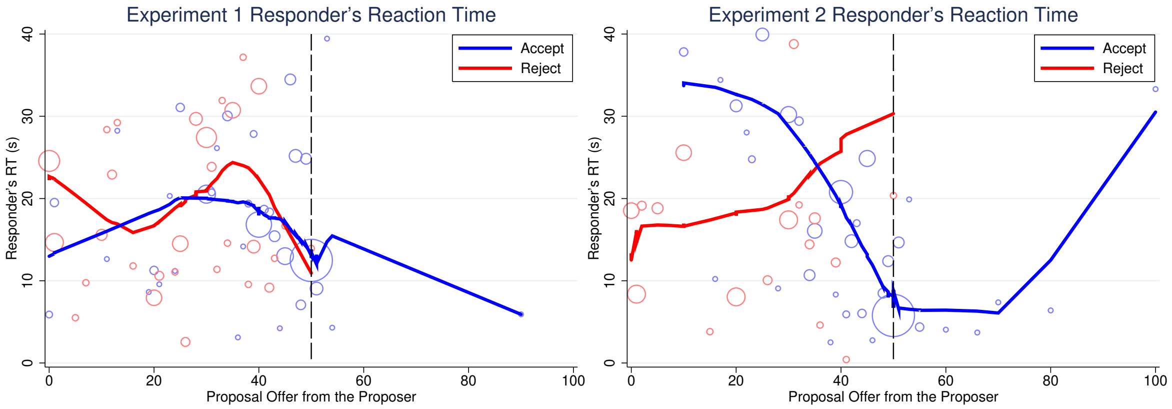

S6 Fig: The LOWESS estimation of and scatter plot of Respnder's reaction time. The size of the bubbles is weighted by the number of observations.

B.5 Risk Elicitation

S14 Table: Risk Elicitation Task

	N	Mean	SD	Max	min
# of safe options					
Experiment 1	545	4.396	2.047	0	9
Experiment 2	468	4.891	2.274	0	9

Note: There are 62 players in Experiment 1 and 91 players in Experiment 2 that are not rationalizable and are excluded from the table and the following analysis.

The fifth task in the playlist was a multiple price list risk elicitation task based on Holt and Laury (2002) (which is referred to as the "dice lottery" game in MobLab). Subjects face 10 choices between two lotteries, labeled Option A or Option B (see Section E.3). The main difference relative to Holt and Laury (2002) is that amounts the amounts at stake for the different options are slightly different.[2] Similar to Holt and Laury (2002), lottery option A in each pair (the "safe option") has a lower variance in prize outcomes. The expected payoff of the safer lottery A is initially greater than that of the high variance lottery choice B, but the probabilities of receiving the two amounts vary systematically with each choice (in the same manner as in Holt and Laury (2002)) so that by the third choice our our experiment (the fourth choice of Holt and Laury (2002)) and continuing through the 10th choice, the expected payoff from the higher variance lottery is always greater than for the low variance, safe lottery. Thus, in our experiment a risk neutral player would choose the low variance lottery A for the first 3 choices and the high variance lottery for the last 7 choices. Departures from this risk neutral prediction specifically, more than (less than) 3 choices in a row of the low variance lottery indicate risk averse (risk loving) preferences with respect to uncertain money amounts. A second difference between our experimental design and that of Holt and Laury (2002) is that we only allow subjects to switch from choice A to choice B one time (it is also possible to switch once from choice B to choice A). This design implements a single-crossing and avoid non-monotonic back and forth switching between the two lottery choices that confounds the analysis of risk attitudes. Finally, following completion of the Holt and Laury task, we only report the amount that subjects earned (in points).

S14 Table and S7 Fig summarize the results of this risk

[2] For option A, the amounts in Holt and Laury (2002) are \$2 and \$1.60 whereas we have \$4 and \$5 while for option B, the amounts in Holt and Laury (2002)are \$3.85 and \$0.10, whereas we have \$10 and \$1.

elicitation task in Experiments 1 and 2. First, we observe that there is only a small amount of risk-seeking players in both Experiment 1 and 2 (11.6% in Experiment 1 and 13.0% in Experiment 2). Moreover, on average, players in both experiments are more risk averse than risk neutral decision makers by 1.395 and 1.891 safe options, which is consistent with the finding in Holt and Laury (2002). Finally, from the right panel of S7 Fig, we can find Experiment 2 players are significantly more risk averse than Experiment 1 players ($KS = 0.1341, p < 0.001$).

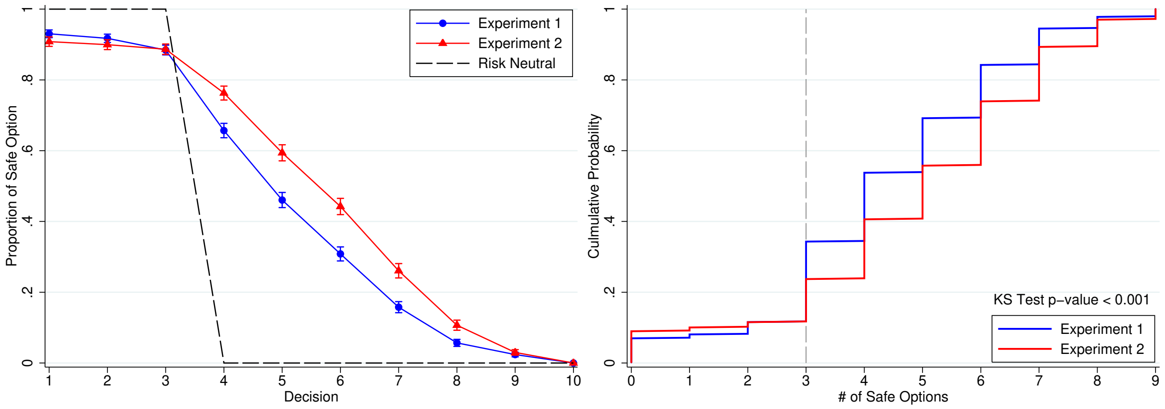

S7 Fig: The left panel shows the average probability of choosing the safe option. The dotted line is the predictions of a risk neutral decision maker. Bars indicate standard error of means. The right panel shows the distribution of number of safe options chosen by players in Experiments 1 and 2. The p-value of a Kolmogorov-Smirnov test is provided in the figure.

B.6 Centipede Game

S15 Table: Centipede Game Summary Statistics

	Experiment 1			Experiment 2		
	N	Mean	SD	N	Mean	SD
Take Rate at Node 1						
Round 1	297	0.283	0.451	272	0.246	0.432
Round 2	307	0.368	0.483	276	0.380	0.486
Round 3	306	0.500	0.501	279	0.527	0.500
Take Rate at Node 2						
Round 1	190	0.426	0.496	193	0.352	0.479
Round 2	182	0.495	0.501	166	0.386	0.488
Round 3	147	0.510	0.502	130	0.515	0.502
Take Rate at Node 3						
Round 1	102	0.637	0.483	117	0.470	0.501
Round 2	91	0.604	0.492	100	0.500	0.503
Round 3	69	0.667	0.475	63	0.444	0.501

The sixth experiment in the playlist was a centipede game, as originally studied by McKelvey and Palfrey (1992). In this two-player game, players 1 and 2 take turns choosing either to "pass" (P) decision-making on to the other player or to "take" (T) the current payoff. See Section E.3 for the game form we implemented in the experiments.

The subgame perfect Nash equilibrium via backward induction is that the first mover, player 1 chooses Take at the first opportunity ending the game with payoffs of 4 points to player 1 and 1 point to player 2. However, this prediction is seldom played. McKelvey and Palfrey (1992) report that only 7% of the first movers chose Take at the first opportunity in a variant of the same four-node game that we study here. The most regular finding in experimental tests of this game with inexperienced subjects is that the frequency of "Take" increases as they move closer to the ending node of the game. A further finding is that experienced subjects, who have played the game at least once, learn to choose Take earlier and earlier in the game with repetition.

S15 Table and S8 Fig summarize the results. The figure shows the frequency with which players chose Take at nodes 1 (Player 1), 2 (Player 2) and 3 (Player 1) in both experiments. This figure reveals that, consistent with the existing experi-mental literature on the centipede game, the frequency of take ("the take rate") is significantly

greater at decision node 2 than at decision node 1 in the first two repetitions of the game. By the third repetition, however, there is no longer any difference in the frequency of take at node 1 or 2, which is the same at 50%. Thus, even after 3 repetitions, only 50% of pairs are playing the subgame perfect Nash equilibrium. The latter frequency is consistent with the findings reported in McKelvey and Palfrey (1992) for the four node centipede game. Interestingly, the take rate at node 1 in our data set of 28.3% is somewhat higher than the 7.1% take rate reported in the four-node game of McKelvey and Palfrey (1992).

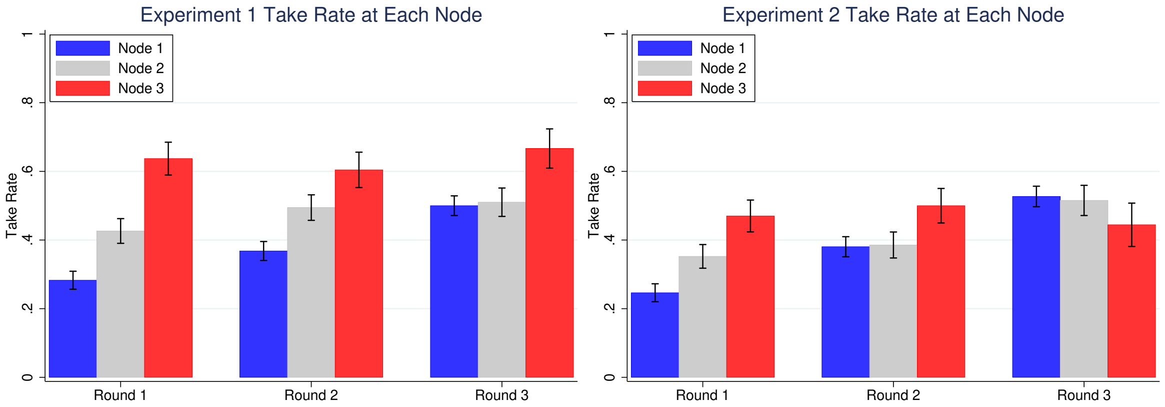

S8 Fig: The take rate at each node (conditional on passing at previous nodes) across three rounds (with standard error bars).

B.7 Trust Game

S16 Table: Trust Game Summary Statistics

	N	Mean	SD	Median	Max	min
Experiment 1						
Investment	308	29.02	30.72	20	100	0
Return Ratio	219	0.602	0.718	0.322	3	0
Experiment 2						
Investment	280	38.48	34.69	25	100	0
Return Ratio	223	0.465	0.604	0.030	3	0

Note: The return ratio is the ratio between amount of return and the amount of investment from player 1. It would be missing if player 1 invests nothing.

The seventh experiment in the playlist was a trust game as originally studied by Berg et al. (1995). In this two-player game, the first mover (investor) is endowed with \$100 (100 points) while the second mover (responder) is endowed with nothing. The investor decides how much of her endowment to "invest" or "send" to the responder. The amount invested (or sent), I, if positive, is then tripled. Finally the responder decides how much of the tripled amount, $3I$, to "return" to the investor, $R \in [0, 3I]$. The payoff to the investor is $100 - I + R$, and the payoff to the responder is $3I - R$. If this game is played once (as in our study), via backward induction the unique subgame perfect Nash equilibrium is for the responder to always return 0 and thus for the sender to always send 0. However, as Berg et al. (1995) first reported, this is not what happens in the one-shot game. They found that investors sent, on average, 51.6% of their \$10 endowment, while the average amount returned by responders was about 90% of the amount invested I (or 30% of the tripled investment amount, $3I$).

We observe similar, but slightly different results in the data from our Xiamen experiment as reported on in S16 Table and S9 Fig. The mean amount invested was 29.02 in Experiment 1 and 38.48 in Experiment 2. Moreover, a Kolmogorov-Smirnov test shows that the distribution of investment in Experiment 1 is significantly different from the distribution in Experiment 2 (KS = 0.1669, $p < 0.001$), indicating the investment in Experiment 2 is larger. The mean ratio of the amount returned to the amount invested is on average, 60.24% in Experiment 1 and 46.53% in Experiment 2 which are less than the 90% average of Berg et al. (1995) and the return rate decreases with the invested amount. Specifically, a no-intercept regression of the return amount on the investment amount, as

plotted in S9 Fig, has a positive and significant slope coefficient of 0.707 ($\hat{\beta} = 0.707$, $t = 7.97$, $p < 0.001$, 95% C.I. = [0.533, 0.882]) for Experiment 1 and 0.680 ($\hat{\beta} = 0.680$, $t = 8.66$, $p < 0.001$, 95% C.I. = [0.526, 0.834]) for Experiment 2.

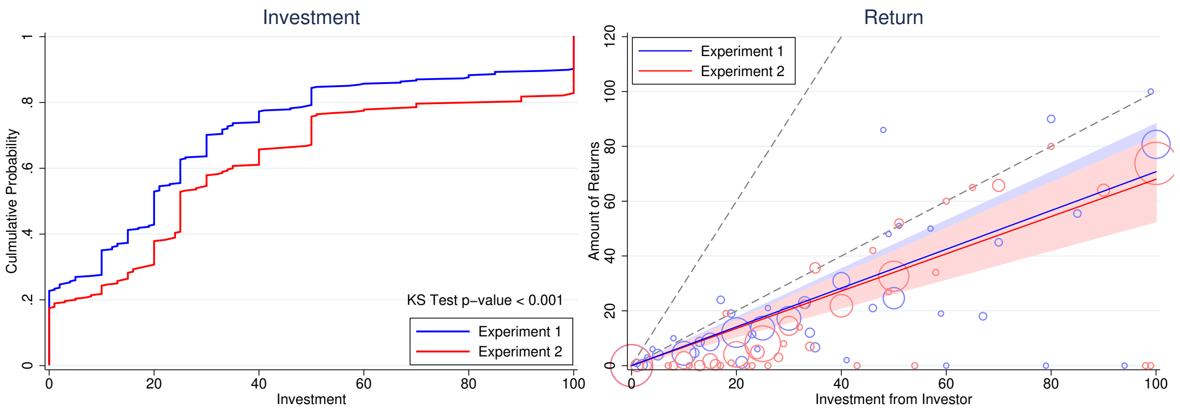

S9 Fig: The left panel shows the cumulative probability function of invest-ment from Experiment 1 and 2. The p-value of the Kolmogorov-Smirnov test is shown in the figure. The right panel shows the amount of returns conditional on the investment from the investor.

C Gender

C.1 Beauty Contest Game

S17 Table: Beauty Contest Game Summary Statistics

	Experiment 1				Experiment 2			
	N	Mean	SD	Median	N	Mean	SD	Median
Initial Guess								
Female	295	36.73	21.39	34.0	417	37.60	21.44	35.0
Male	112	26.05	19.02	22.5	119	33.08	19.15	33.0

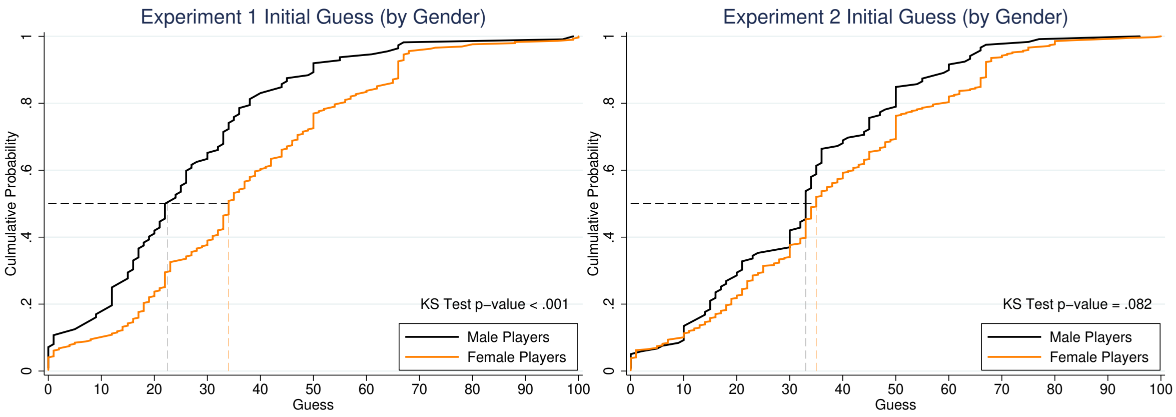

S10 Fig: Cumulative density function of initial guesses divided by gender. The median guesses of large and small groups are labeled in dashed lines. The p-value of the Kolmogorov–Smirnov test is shown at the bottom of each figure.

In this section, we consider gender differences in the initial guess of the beauty contest game. Gender differences have been the focus of many experimental studies (see, e.g., Croson and Gneezy (2009)), and so we will also consider the evidence for gender differences in several of the games of our experiment as well. We focus on initial guess in the beauty contest game, as that is the only period for which decisions are not affected by feedback on the choices made by others and thus provides the cleanest test of gender differences. For this same reason, we will also use the first period beauty contest guesses to classify players in terms of their strategic sophistication as well (see Supplementary Section D.1).

S17 Table reports the summary statistics and S10 Fig shows male and female players' CDFs. From the Figure, we can observe that in Experiment

1, the females' initial guesses tend to be higher (further away from the equilibrium) than the males'. A Kolmogorov-Smirnov test confirms that the distribution of initial guesses by females and males is not the same ($KS = 0.2620, p < 0.001$) favoring the alternative view that female guesses are higher, on average. Yet, in Experiment 2, the null hypothesis can only be rejected at $p = 0.1$ significant level ($KS = 0.1267, p = 0.082$).

This finding is consistent with some, but not all studies exploring gender differences in beauty contest games. For instance, Cubel and Sanchez-Pages (2017) find that males employ more steps of iterated reasoning than do females in BC games without monetary incentives, but these differences disappear with monetary incentives. Perhaps more relevant to our findings, Qin (2020) repeats the design of Cubel and Sanchez-Pages (2017) on Chinese university students and finds that males employ greater steps of iterated reasoning than do females, even *with* monetary incentives.

C.2 Ultimatum Game

S18 Table: Ultimatum Game Summary Statistics

	Experiment 1			Experiment 2		
	N	Mean	SD	N	Mean	SD
Proposal Offer						
Female	235	37.56	14.29	211	37.73	14.75
Male	77	31.94	18.07	59	35.34	18.35
Acceptance Rate						
Female	225	0.711	0.454	207	0.792	0.407
Male	77	0.662	0.476	58	0.741	0.442

Here we analyze the gender difference in the ultimatum game. S18 Table reports the summary statistics in both Experiments 1 and 2. Moreover, S11 Fig shows the distribution of proposal offers and the conditional acceptance rates (conditional on the proposal offers from the proposer).

First of all, we can see the left panels of S11 Fig that male proposers offer significantly less than female proposers in Experiment 1 (KS Test: $KS = 0.1822$, $p = 0.030$) but not in Experiment 2 (KS Test: $KS = 0.0994$, $p = 0.695$). In other words, the result of the KS test implies that females in the first mover role offer more in Experiment 1 but there is no gender difference in Experiment 2.

The right panels of S11 Fig plot the conditional acceptance rates for

males and females. In Experiment 1, we can observe that for the conditional acceptance rate, females and males have similar slopes before 50 ($\hat{\beta}_{Male} = 0.014, t = 4.02, p < 0.001, 95\%$ C.I. $=$ $[0.007, 0.021]$ and $\hat{\beta}_{Female} = 0.018, t = 8.58, p < 0.001, 95\%$ C.I. $= [0.014, 0.022]$). Also, both females and males tend to accept any offers above 50.

Experiment 2 replicates this observation. We find that females and males have similar slopes before 50 ($\hat{\beta}_{Male} = 0.023, t = 6.96, p < 0.001, 95\%$ C.I. $= [0.017, 0.030]$ and $\hat{\beta}_{Female} = 0.019, t = 10.55, p < 0.001, 95\%$ C.I. $= [0.015, 0.022]$). Finally, we also observe that both females and males would accept any offers above 50. This result shows that there is no gender difference in conditional acceptance rates in both Experiments 1 and 2.

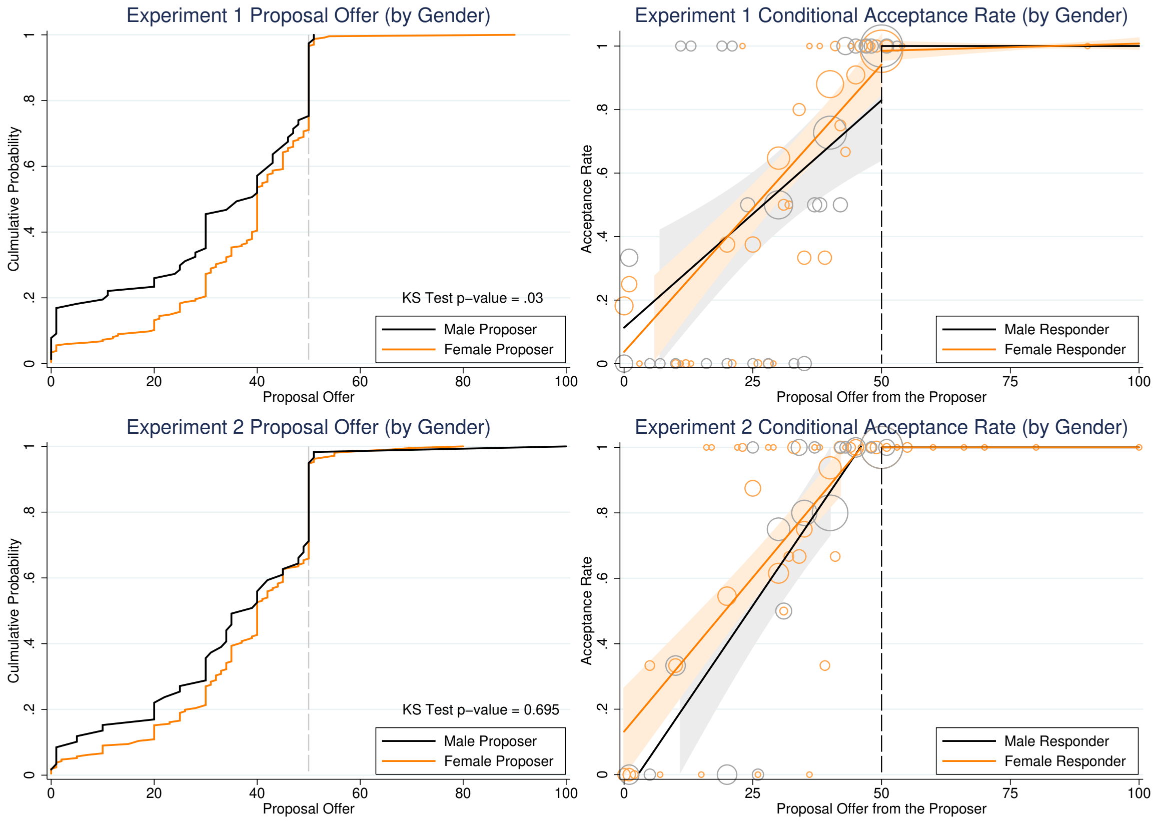

S11 Fig: Proposal offers and acceptance rates divided by gender.

C.3 Risk Elicitation Task

In this section, we analyze whether there is any gender difference in risk preference in our data set. The average number of safe options in each case is reported in S19 Table and the distributions are shown in S12 Fig

S19 Table: Risk Preference Summary Statistics

	Experiment 1			Experiment 2		
	N	Mean	SD	N	Mean	SD
# of safe options						
Female	403	4.467	2.066	348	5.144	2.217
Male	142	4.197	1.987	105	4.095	2.356

In Experiment 1, a KS test of the null hypothesis of no difference in the distribution of choices between males and females cannot be rejected ($KS = 0.0693, p = 0.651$). However, the null hypothesis is rejected in Experiment 2 ($KS = 0.2180, p = 0.001$). Our finding from Experiments 1 and 2 is consistent with the mixed evidence that has been found for gender differences in risk aversion using multiple price lists and other tasks, see, e.g., Filippin and Crosetto (2016).

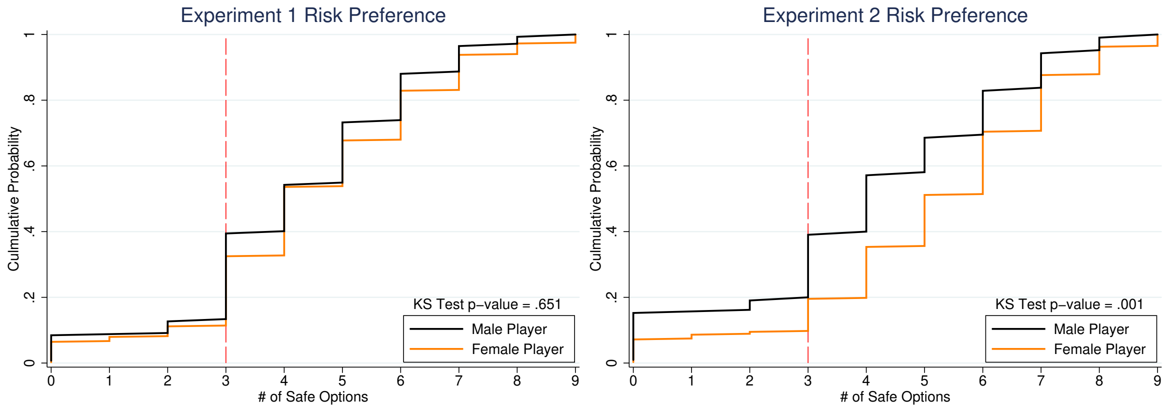

S12 Fig: The distribution of number of safe options chosen by male and female players in Experiment 1 and 2.

C.4 Centipede Game

S20 Table: Centipede Game Summary Statistics

	Experiment 1 Male			Experiment 1 Female			Experiment 2 Male			Experiment 2 Female		
	N	Mean	SD	N	Mean	SD	N	Mean	SD	N	Mean	SD
Take Rate at Node 1												
Round 1	78	0.321	0.470	218	0.266	0.443	50	0.200	0.404	210	0.238	0.427
Round 2	82	0.390	0.491	224	0.357	0.480	51	0.373	0.488	213	0.380	0.487
Round 3	81	0.543	0.501	224	0.482	0.501	52	0.442	0.502	215	0.540	0.500
Take Rate at Node 2												
Round 1	44	0.477	0.505	146	0.411	0.494	46	0.283	0.455	142	0.366	0.483
Round 2	38	0.553	0.504	144	0.479	0.501	39	0.308	0.468	125	0.416	0.495
Round 3	36	0.472	0.506	111	0.523	0.502	38	0.526	0.506	90	0.522	0.502
Take Rate at Node 3												
Round 1	31	0.645	0.486	71	0.634	0.485	27	0.481	0.509	85	0.459	0.501
Round 2	21	0.619	0.498	70	0.600	0.493	19	0.421	0.507	78	0.526	0.503
Round 3	18	0.611	0.501	51	0.686	0.469	15	0.267	0.458	47	0.511	0.505

This section investigates whether there is any gender difference in the centipede game. First of all, the summary statistics are reported in S20 Table and the data are visualized in S13 Fig.

From the data, we can observe that the patterns are similar in Experiments 1 and 2 and for both male and female players. We find that in both Experiments 1 and 2, the players are more likely to choose "Take" earlier in later rounds. If we disaggregate by gender as is done in the S13 Fig, we can find by the third round there is no difference in the take frequencies at nodes 1 and 2 between males and females, with both rates being approximately 50%.

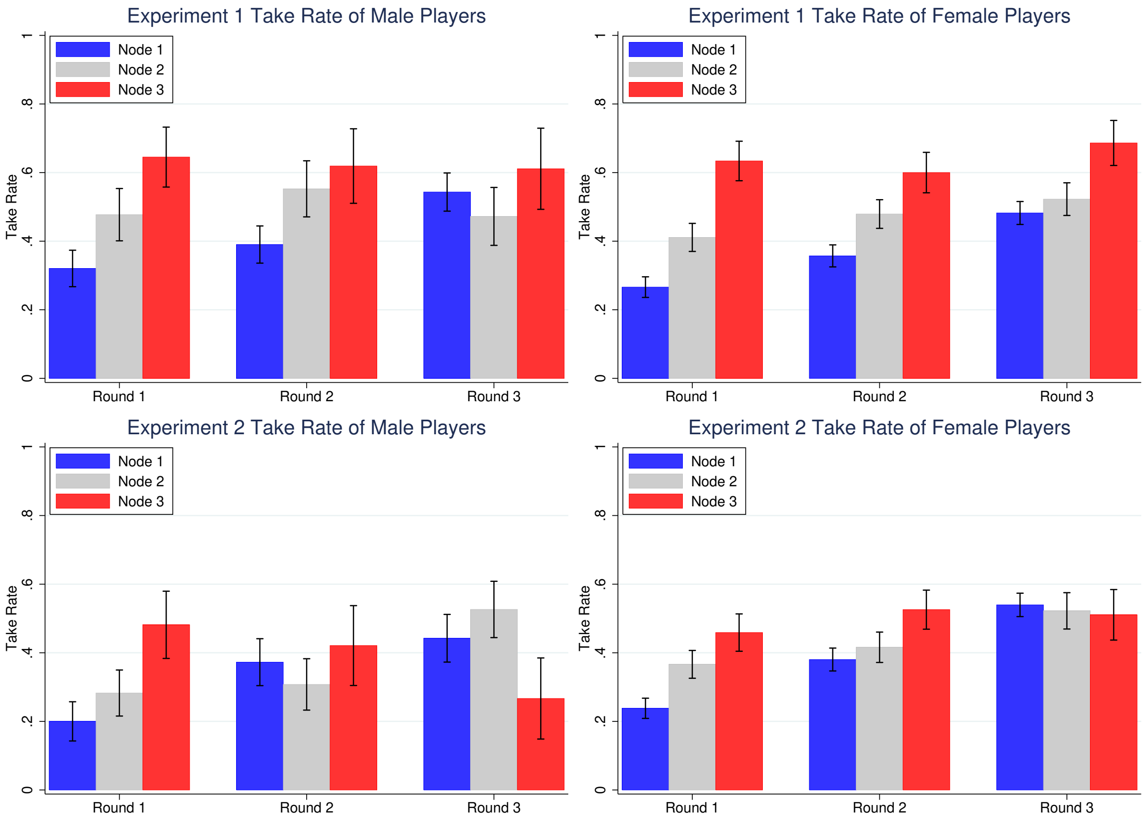

S13 Fig: The take rate at each node (conditional on passing at previous nodes) across three rounds (with standard error bars) for male and female players.

C.5 Trust Game

S21 Table: Trust Game

	Experiment 1			Experiment 2		
	N	Mean	SD	N	Mean	SD
Investment						
Female	227	24.81	25.96	215	34.66	32.43
Male	81	40.84	39.08	62	52.19	39.59
Return Ratio						
Female	166	0.564	0.633	166	0.457	0.601
Male	52	0.737	0.938	45	0.486	0.649

Note: The return ratio is the ratio between amount of return and the amount of investment from player 1. It would be missing if player 1 invests nothing.

In this section, we explore the gender difference in the trust game. First of all, S21 Table reports the summary statistics. We can observe that in both Experiments 1 and 2, male investors tend to invest more than female players while there is no gender difference in return.

S14 Fig also reveals evidence of gender differences in Investment deci-sions. In Experiment 1, we find that female investors invest less, on average 24.81, while male investors invest on average more, 40.84. A Kolmogorov-Smirnov test reveals that we can reject the null hypotheses ($KS = 0.2612$, $p < 0.001$) that the distribution of investment amounts by gender is the same in favor of the alternative that females invest less. Similarly, we find that a Kolmogorov-Smirnov test can reject the null hypotheses ($KS = 0.2854$, $p < 0.001$) in Experiment 2.

As for the return rate, there appears to be no gender difference here, as the estimated regression lines of return on investment are essentially the same for females and males. In Experiment 1, the estimated slope for male player 2 is 0.696 ($\hat{\beta} = 0.696, t = 3.41, p = 0.001, 95\%$ C.I. $= [0.293, 1.099]$) while the slope for female player 2 only differs by 0.016 ($t = 0.07, p = 0.944, 95\%$ C.I. $= [-0.425, 0.456]$). On the other hand, we find a similar pattern in Experiment 2. The estimated slope for male player 2 is 0.650 ($\hat{\beta} = 0.650, t = 3.77, p < 0.001, 95\%$ C.I. $= [0.310, 0.989]$) while the slope for female player 2 only differs by 0.060 ($t = 0.30, p = 0.762, 95\%$ C.I. $= [-0.327, 0.446]$).

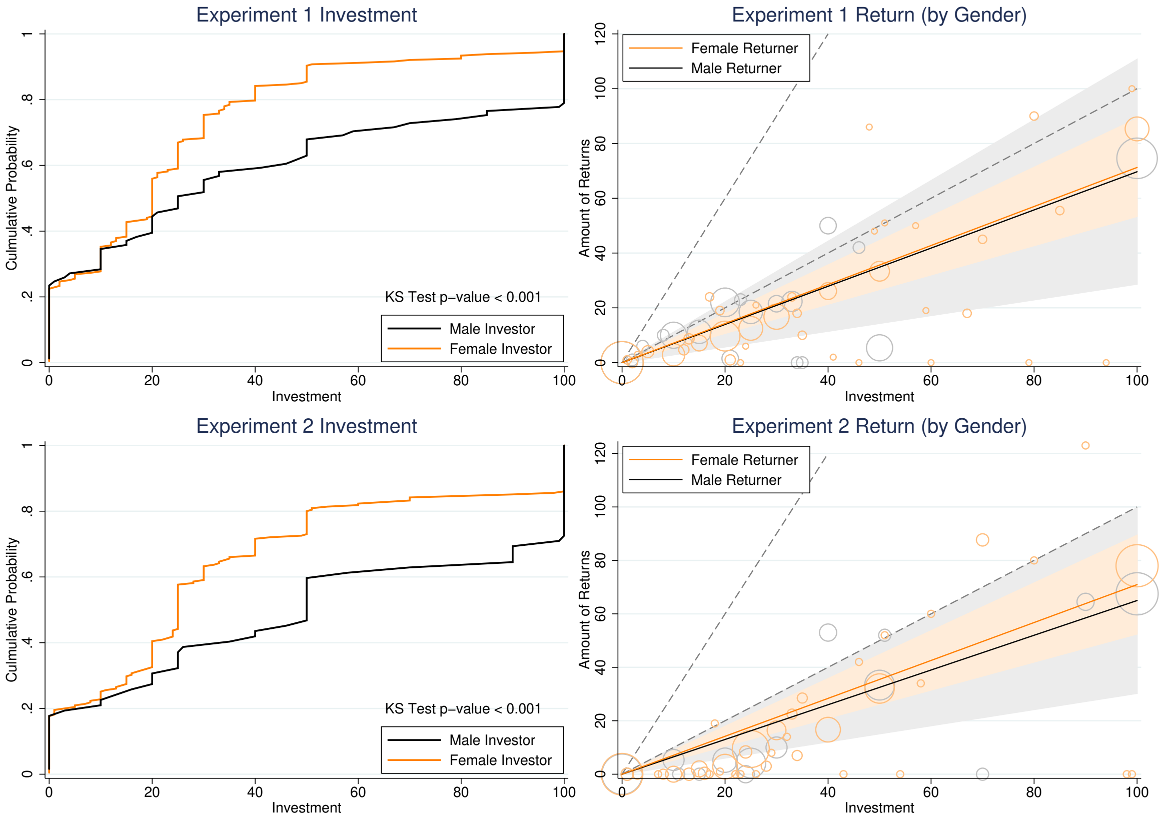

S14 Fig: The left panels show the cumulative probability functions of invest-ment for male and female players. The p-value of the Kolmogorov-Smirnov test is shown in the figure. The right panels show the amount of returns conditional on the investment from the investor.

C.6 Math Competition

The final experiment in the playlist was a game exploring gender differences in the choice between piece-rate and tournament compensation schemes as originally studied by Niederle and Vesterlund (2007). Here we implemented a MobLab variant of this game called "Math Competition."

In the first stage of this 3-stage, individual choice game, subjects have three minutes to solve as many addition puzzles as they can. In each puzzle, they are presented with nine, two-digit numbers and they have to find (click on) the two numbers among the nine that exactly add up to 100. In this stage, they get 1 point per correct answer, and so this stage is known as the "piece rate" compensation scheme.

They then move on to a second stage where face the same, 3-minute task of solving the same type of addition puzzles as in the first stage, but in this second stage they compete with three other robot players. These robot players are programmed to mimic the play of actual human subjects in terms of the number of addition puzzle tasks they can complete in 3 minutes. Thus, this second stage comprises the "tournament" compensation scheme. The human subject gets the same number of points per correct answer as in the piece rate treatment, and receives 4 times this number of points, but only if he/she wins the tournament, that is, if he/she solves more puzzles in the three minute time period than do the three other robot players. Otherwise earnings are 0 in the tournament scheme.

Finally, prior to the third and final stage, subjects have to decide whether or not they want the third stage of this task to be the piece rate or the tournament environment. This choice is the primary outcome variable of interest to researchers conducting such experiments. After making their binary choice (piece rate or tournament), subjects then participate in 3 minutes of their chosen task with the same incentive structures as described earlier.

The main finding from a similar study by Niederle and Vesterlund (2007) is that while there is no difference in performance between men and women in the piece rate or the tournament compensation settings, when given a choice, women disproportionately prefer the piece rate compensation scheme while men prefer the tournament scheme. Specifically, they find that 73 percent of men but only 35 percent of women choose the tournament. While our experiment differs in several respects from Niederle and Vesterlund (2007)[3], we nevertheless obtain qualitatively similar findings as reported on in S15 Fig.

[3] They compared balanced groups of 2 women and 2 men while our experiment is an individual choice task, where subjects play against robot players in the tournament. Our addition task of choosing two numbers out of nine that sum to 100 is different from their task of of adding up adding up as many sets of five two-digit numbers in five minutes as possible. Furthermore, in contrast to Niederle and Vesterlund (2007), our subject pool is not gender-balanced.

In Experiment 1, we find that 62.2% ($N = 156$, S.D. $= 0.487$) of male players choose the tournament while only 46.4% ($N = 463$, S.D. $= 0.499$) for female players (Mann-Whitney Rank-sum Test: $p < 0.001$). Further, if we break down the tournament choice according to whether the player won or lost the tournament in stage 2, we observe that conditional on *losing* the tournament men are significantly more likely to choose the tournament than are women ($p < 0.001$). However, conditional on *winning* the tournament, we find no gender difference in the willingness to choose the tournament ($p > 0.10$). In addition, we find similar results in Experiment 2 where 52.9% ($N = 119$, S.D. $= 0.501$) of male players choose the tournament while 39.8% ($N = 420$, S.D. $= 0.490$) for female players (Mann-Whitney Rank-sum Test: $p = 0.010$). Also, conditional on winning the tournament, we do not find any gender difference ($p = 0.780$) but males players are more likely to choose tournament than female players conditional on losing in the second stage ($p = 0.057$).

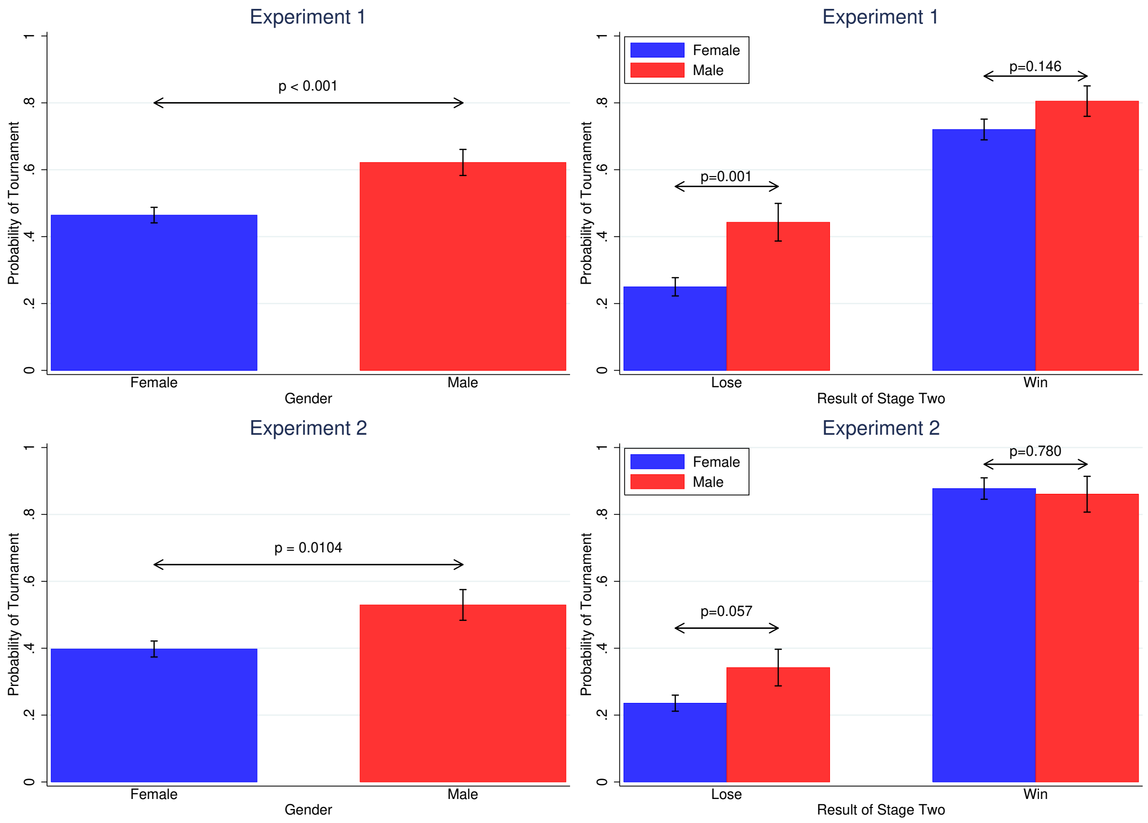

S15 Fig: The left panels show the probability of choosing tournament for female and male players. The right panels show the probability of choosing the tournament conditional on the result of stage two. The p-values of Mann–Whitney rank-sum tests are provided in the figures.

D Behavior Across Games

Over the past three decades, behavioral and experimental economists have made huge progress in identifying the stylized facts of these games (see Camerer (2003)). However, an empirical understanding of how strategic behavior varies across these games remains an open question.

The multi-game protocol of our experiment allows us to observe each player's strategic profile across many different games and hence to look for an empirical relationship in strategic behavior between games. If a player follows the Nash Equilibrium, then he might believe his opponents are also rational and best respond to such a belief. Moreover, he has to believe his opponents would also best respond to a rational strategic profile so that the choices and beliefs are consistent in equilibrium, yielding perfect behavioral correlations across games.

Reaching an equilibrium requires that all players behave fully rationally. If this is not the case, players may choose non-equilibrium strategies and correlations in behavior across games are weakened. For instance, Agranov et al. (2012) and Alaoui and Penta (2016) suggest that observed strategic sophistication are not only determined by the reasoning ability of subjects but also by their beliefs about opponents' sophistication. Psychological factors other than bounded rationality, such as social preferences, can also drive players to deviate from the equilibrium. However, it is not clear if there is any association in dis-equilibrium behavior across different games. Here we take advantage of our design to investigate correlations in strategic measures across games or attributes.

Specifically, we use two approaches to analyze these correlations. First, we explore how an individual's sophistication level, as classified by their first guesses in the p-beauty contest game (also their first choice in the experiment), is correlated with their behavior in the other seven games by a subsample analysis. Second, we compute pairwise Spearman's rank-order correlation coefficients of raw choices in each of the eight games/tasks to summarize the empirical relationship across all games.

D.1 Subsample Analysis

Using the different level type classifications, we report on the mean and 95% CI of various choice and other variables disaggregated by the level type classified by players' initial guesses (see S3 Fig). The sixteen variables that we analyze in this section are sum-marized in S4 Table. In S16 Fig, we look at the relationship between personal characteristics and level types. For instance, in the first panel, we look at how the mean and standard error of the standardized Gaokao score varies with level type. We can observe that see higher levels tend to have higher Gaokao scores in Experiment 2

(Non-parametric trend test: $p = 0.033$) but not in Experiment 1 (Non-parametric trend test: $p = 0.205$). In addition, the only personal characteristic displaying a clear trend in Experiment 1 is gender (Non-parametric trend test: $p < 0.001$), indicating the proportion of female players is smaller in higher levels.

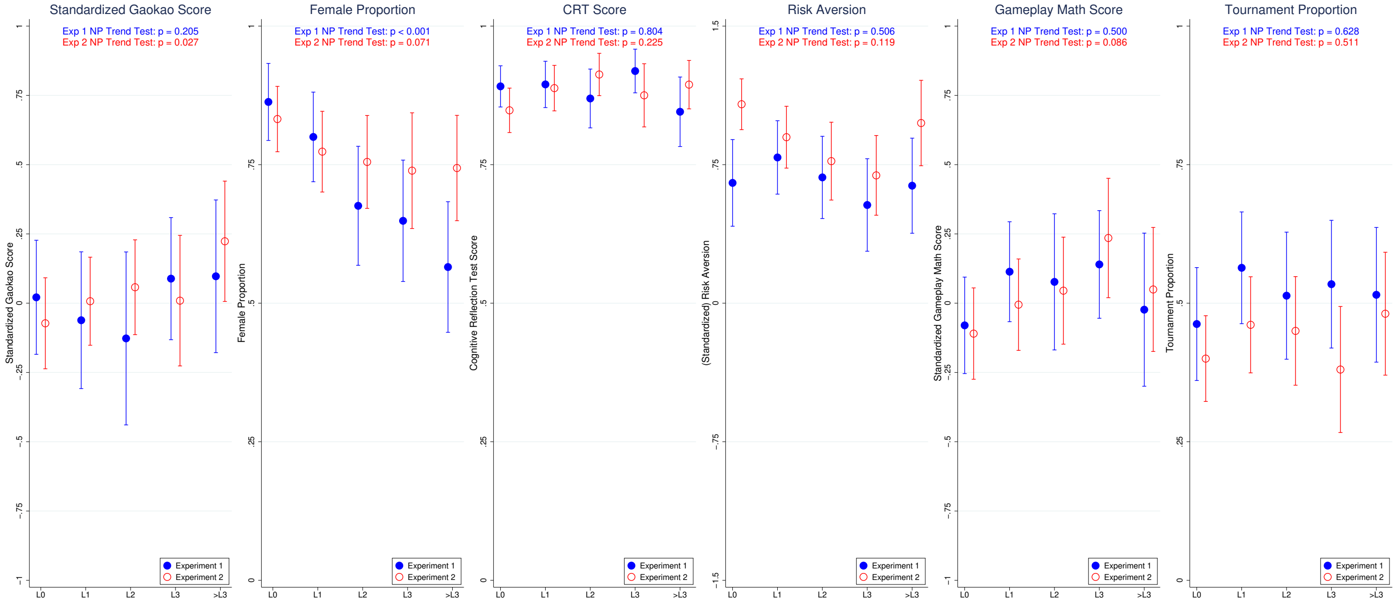

S16 Fig: Mean of basic variables for each level in the beauty contest game. Bars indicate the 95% CI. The p-values of non-parametric trend tests are provided in the figures.

S17 Fig reports on the association between various strategic choices and beauty contest levels using the same format as in S16 Fig. Here we find several interesting and significant trends. In Experiment 1, we find that higher level players are (1) more likely to make voter turnout choices according to the Bayesian Nash Equilibrium (BNE) prediction (Non-parametric trend test: $p = 0.018$), (2) more likely to perform strong free-riding behavior (Non-parametric trend test: $p < 0.001$), and (3) more likely to offer less in the ultimatum game (Non-parametric trend test: $p = 0.003$). On the other hand, in the Experiment 2, we also find higher level players are more likely to perform free-riding behavior and offer less in the ultimatum game.

Broadly speaking, the monotonic trends are similar across experiments, suggesting that the underlying cause of the correlations is immune to the experimental population. Focusing on the strategic measures with significant trends, we find that the pattern is consistent with the direction of Nash equilibrium—more sophisticated players are more likely to choose the

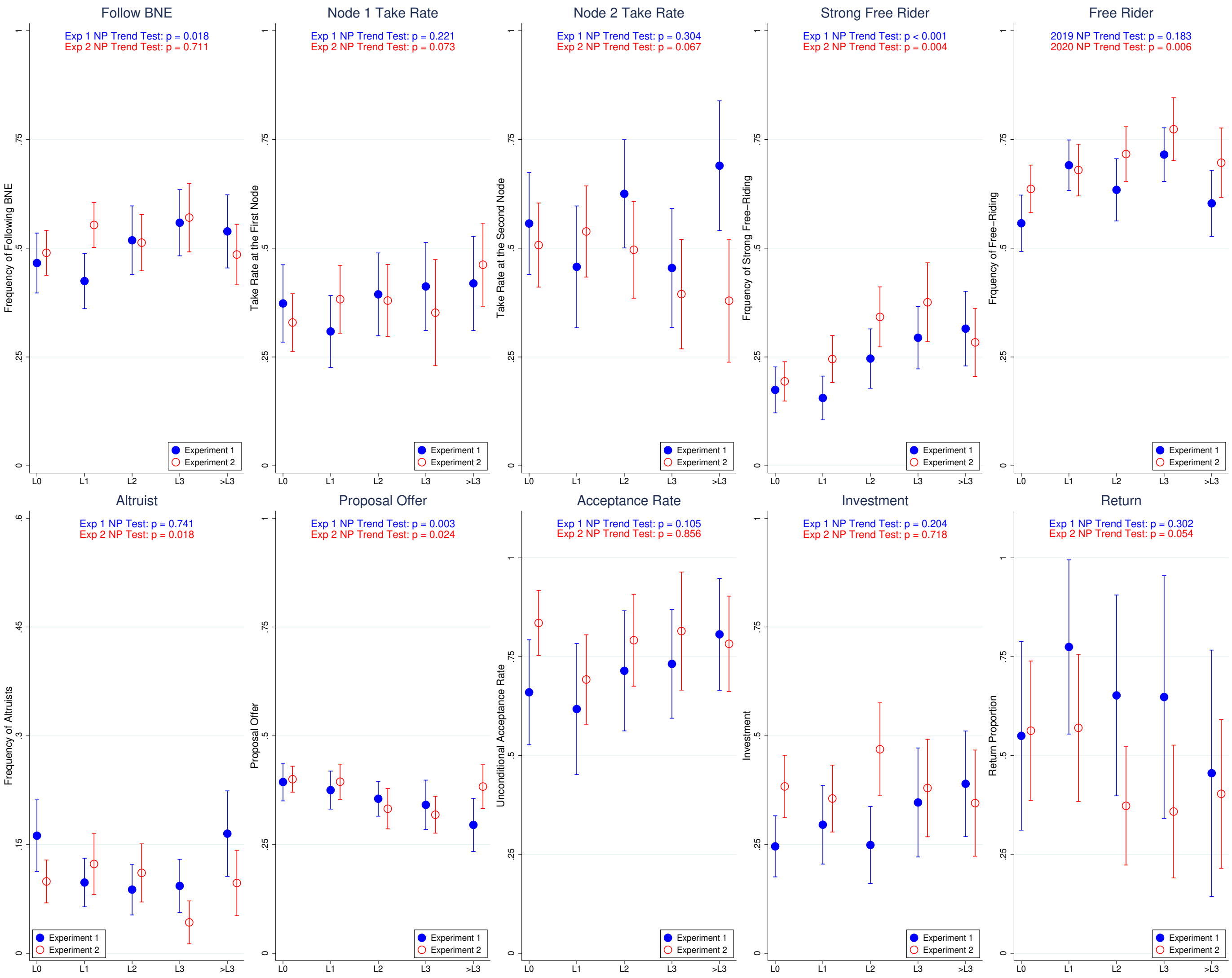

S17 Fig: Mean of strategic behavior for each level in the beauty contest game. Bars indicate the 95% CI. The p-values of non-parametric trend tests are provided in the figures.

strictly dominant strategy of contributing nothing in the public goods game and their offers are closer to the subgame perfect equilibrium in the ultimatum game. This demonstrates the predictive power of the equilibrium while its sensitivity to the beliefs about other players' behavior.

D.2 Spearman's Rank-Order Correlation Coefficients

Experiment 1 Spearman Correlation Matrix

	Risk Aversion	Initial Guess	Follow BNE	Take Rate at Node 1	Strong Free Rider	Proposal Offer	Investment
Risk Aversion	1.0000						

Initial Guess	0.0276	1.0000					
	[1.0000]	---					
Follow BNE	0.0109	-0.1056	1.0000				
	[1.0000]	[0.7108]	---				
Take Rate at Node 1	-0.0232	-0.0979	0.0168	1.0000			
	[1.0000]	[1.0000]	[1.0000]	---			
Strong Free Rider	-0.1380	-0.2129	0.0989	0.1196	1.0000		
	[0.0261]	[0.0003]	[0.3197]	[0.7412]	---		
Proposal Offer	0.0002	0.2046	-0.0644	-0.0437	-0.1606	1.0000	
	[1.0000]	[0.0637]	[1.0000]	[1.0000]	[0.0923]	---	
Investment	-0.0329	-0.0645	0.0436	-0.1642	-0.0571	-0.0465	1.0000
	[1.0000]	[1.0000]	[1.0000]	[0.8931]	[1.0000]	[1.0000]	---

Positively Correlated: [p<0.01] [p<0.05] [p<0.10] — Uncorrelated — Negatively Correlated: [p<0.10] [p<0.05] [p<0.01]

Experiment 2 Spearman Correlation Matrix

	Risk Aversion	Initial Guess	Follow BNE	Take Rate at Node 1	Strong Free Rider	Proposal Offer	Investment
Risk Aversion	1.0000						

Initial Guess	0.0610	1.0000					
	[1.0000]	---					
Follow BNE	-0.0051	-0.0163	1.0000				
	[1.0000]	[1.0000]	---				
Take Rate at Node 1	0.0684	-0.1075	0.0902	1.0000			
	[1.0000]	[1.0000]	[1.0000]	---			
Strong Free Rider	-0.1713	-0.1458	0.0893	0.1152	1.0000		
	[0.0011]	[0.0136]	[0.7676]	[1.0000]	---		
Proposal Offer	0.1524	0.1436	-0.0755	0.0442	-0.2076	1.0000	
	[0.2358]	[0.3688]	[1.0000]	[1.0000]	[0.0106]	---	
Investment	-0.1062	0.0048	0.0334	-0.2472	0.0016	0.0547	1.0000
	[1.0000]	[1.0000]	[1.0000]	[0.0755]	[1.0000]	[1.0000]	---

Positively Correlated: [p<0.01] [p<0.05] [p<0.10] — Uncorrelated — Negatively Correlated: [p<0.10] [p<0.05] [p<0.01]

S18 Fig: Pairwise Spearman's rank-order correlation coefficients of strategic behavior. Color-coded correlation coefficients indicate significance with Bonferroni corrections. We use warm colors for positive correlations and cold colors for negative correlations. A darker color implies the correlation coefficient is more statistically significant. The left panel shows the correlation matrix for Experiment 1 and the right panel is for Experiment 2.

S18 Fig summarizes the pairwise Spearman's rank-order correlation co-efficients for the 7 measures used in the prior section. We apply the Bonferroni correction to counteract the problem of multiple comparisons for the significance testing of the correlation coefficients.

Similar to the subsample analysis, we observe a significant negative correlation between the initial guess and strong free-riding behavior (Experiment 1: $\rho = -0.2129$, $p = 0.0003$; Experiment 2: $\rho = -0.1458$, $p = 0.0136$). Moreover, proposers who offer less in the ultimatum game are more likely to be a strong free-riders (Experiment 1: $\rho = -0.1606$, $p = 0.0923$; Experiment 2: $\rho = -0.2076$, $p = 0.0106$). This result suggests that the players tend to adopt similar reasoning processes in the beauty contest game, the public good game and the ultimatum game—which is consistently in the direction of the Nash equilibrium.

Furthermore, S18 Fig shows that the more risk averse players are signifi-cantly less likely to be strong free riders (Experiment 1: $\rho = -0.1380$, $p = 0.0261$; Experiment 2: $\rho = -0.1713$, $p = 0.0011$). This significant correlation is unexpected from the perspective of equilibrium since being a strong free-rider is a strictly dominant strategy. That is, a payoff maximizing player should choose this *regardless* of his risk preference. This finding suggests that while Nash equilibrium has predictive power in understanding dis-equilibrium behavior, it cannot be the sole explanation. Alternatively, models with social image concerns can support

such an empirical relationship if players believe there is a non-trivial probability that their behavior is “observed”—viz. the “audience effect” (see Andreoni and Bernheim (2009)).

Finally, the insignificant correlation between the risk preference measure and the amount of investment in the trust game is consistent with the finding in Dean and Ortoleva (2019). Conceptually, sending money to another player in the first stage of the trust game is an uncertain prospect and hence the correlation between investment and risk aversion is plausible. Yet, the insignificance indicates the players in the trust game do not view the investment as a gamble. Instead, the weak correlation between the investment amount and the take rate in the centipede game that we found in Experiment 2 ($\rho = -0.2472$, $p = 0.0755$) suggests that investment behavior is potentially related to the belief forming ability in a multi-stage game.

E Method

In this section, we first provide some general information about the participants and the experimental implementation. In addition, we provide the instruction slides and screenshots of all games in the second subsection. Finally, the details of game configurations are reported in the last subsection.

E.1 Background of Participants and Experimental Implementation

For both Experiment 1 and 2, subjects were recruited from a group of college students participating in an economic summer camp at Xiamen University. Participants were students who just completed their third year of university study and wanted to go to graduate school for a master or Ph.D degree. They applied with references from their own university and were selected by the School of Economics at Xiamen University for a 5-day summer camp. The summer camp includes the first 2-day lectures by faculty members to introduce all the fields in economics at Xiamen University and the following 3-day exams for the qualification of entering the graduate programs of economics at Xiamen University without taking the national entry exams for graduate schools. Due to COVID-19, the summer camp in 2020 and the experiment were held online.

Both Experiment 1 and Experiment 2 were conducted through MobLab which is an online educational platform for conducting economics experiments. The eight games we used in the experiment were pre-programmed by MobLab. Experiment 1 was a large scale in-person experiment which was conducted as one of the 2-day lectures for a duration of 2 hours on the morning of the first day of the summer camp. On the other hand, Experiment 2 was conducted online. They were notified that their performance in the experiment would not affect the evaluation of their qualification.

Students were asked to install the app of the online platform (MobLab) and register for the experiment on their phone two days before the actual experiment session. A welcome survey on the app was conducted one day before the experiment session to confirm the installation and registration. There were 633 players who at least participated in one of the eight games in Experiment 1 and 585 players in Experiment 2.

Experiment 1 began with a 15-minute introduction to experimental economics and the experimental lab and the research group at Xiamen University, followed by a 2-hour session of online experiment in an auditorium with a capacity of 800 seats. The experiment instructor explained the games one by one using slides (see section E.2) on two large screens and there were 18 well trained research assistants each in charge of one section of auditorium to help

students with any technical or game related questions during the experiment. The 18 research assistants were undergraduate or graduate students at Xiamen University and had been trained in three one-hour long sessions by MobLab team members before the experiment.

Experiment 2 was an online experiment. The experimenter explained the instructions via Dingtalk which was a communication and collaboration platform which supports large-scale video conferences. The subjects can start joining the live video conference on Dingtalk 30 minutes before the experiment. During the experiment, the experimenter shared the screen for the experimental instruction and followed the same procedure as Experiment 1. There were 24 well-trained research assistants online helping students with any technical or game related questions during the experiment.

At the beginning of both experiments, we communicated to subjects that their decisions and corresponding points earned from all games would be incorporated into their final payment. They were told to expect a show-up payment of 10 CNY for participating in the experiment. The average payoff per game was 3 CNY with a final payment being the sum of their show-up payment and total payoff across all 8 games. In Experiment 1 and 2, the overall average total payment was 37.61 CNY ($\approx$ 5.42 USD) and 40.00 CNY ($\approx$ 5.77 USD), respectively, or roughly the equivalent of 2 hours of work as an TA in China. Subjects were paid on Alipay, the payments platform of Alibaba, which is ubiquitous in China and is also the world's largest mobile payment platform. Their account information was collected before the experiment with consent and was only used for this experiment.

E.2 Instruction Slides

In this section, we provide the instruction slides and screenshots of each stage in all games.

S19 Fig shows the instructions of the beauty contest game. The experi-menter first explains the rule with S19A Fig, and then introduces the game interface with S19B-S19D Fig. Similarly, the experimenter explains the rest of the games with S20 Fig to S26 Fig.

The experimenter provided each instruction at the beginning of that game. The subjects are told that their final reward depends on their performance in all games and the they will be paid through Alipay after the experiment.

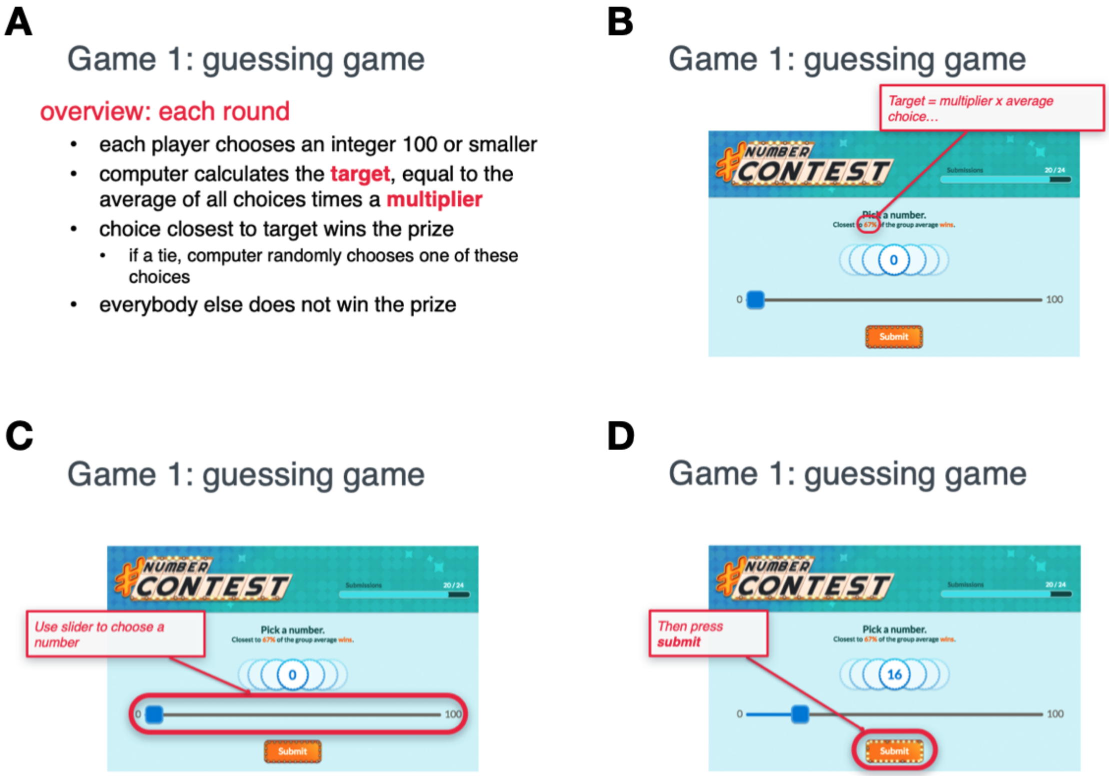

S19 Fig: Instruction slides and screenshots of the beauty contest game. (A) Overview of the rules. (B) Player's initial screen. (C) shows that the decision can be made by moving the slider. (D) reminds the players to click the "submit" bottom to submit their decision.

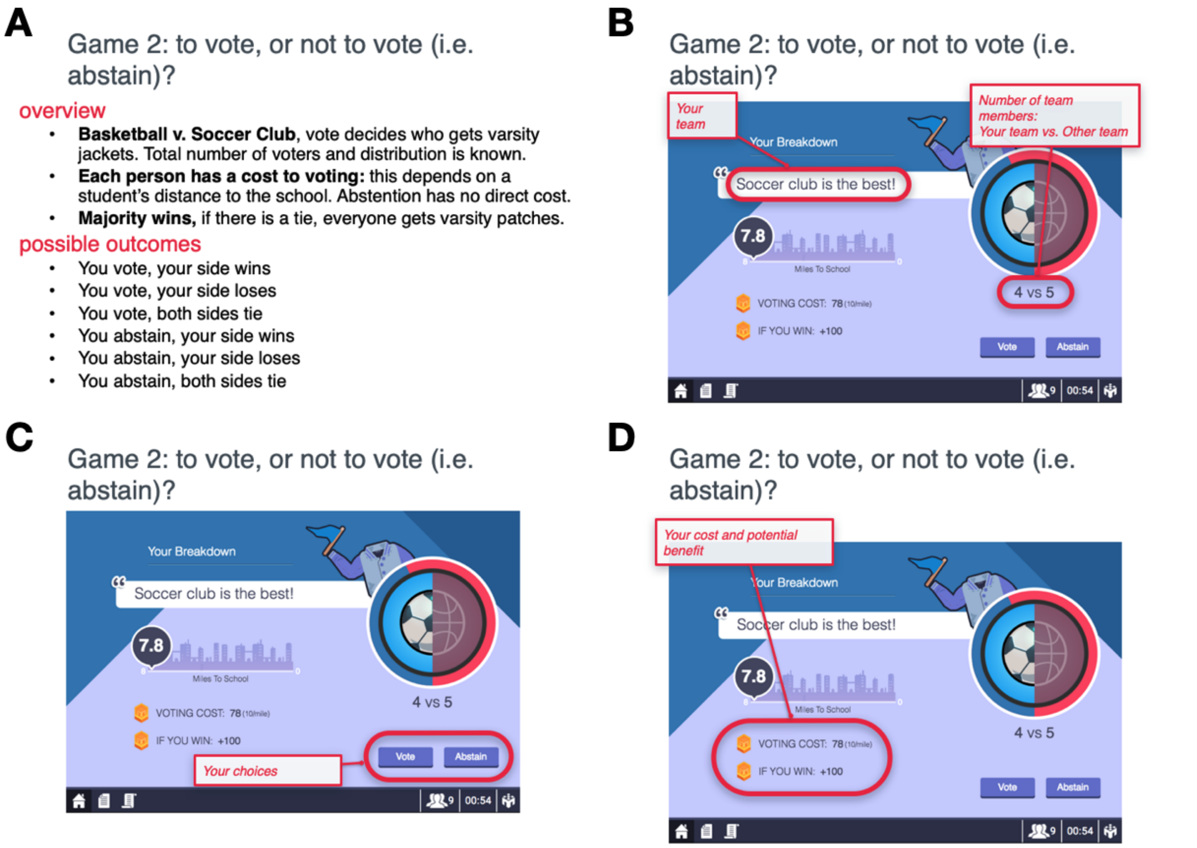

S20 Fig: Instruction slides and screenshots of the voter turnout game. (A) Overview of the rules. (B) Player's initial screen. (C) shows that the players can either "vote" or "abstain." (D) The cost of voting and the payoff are provided in the screen.

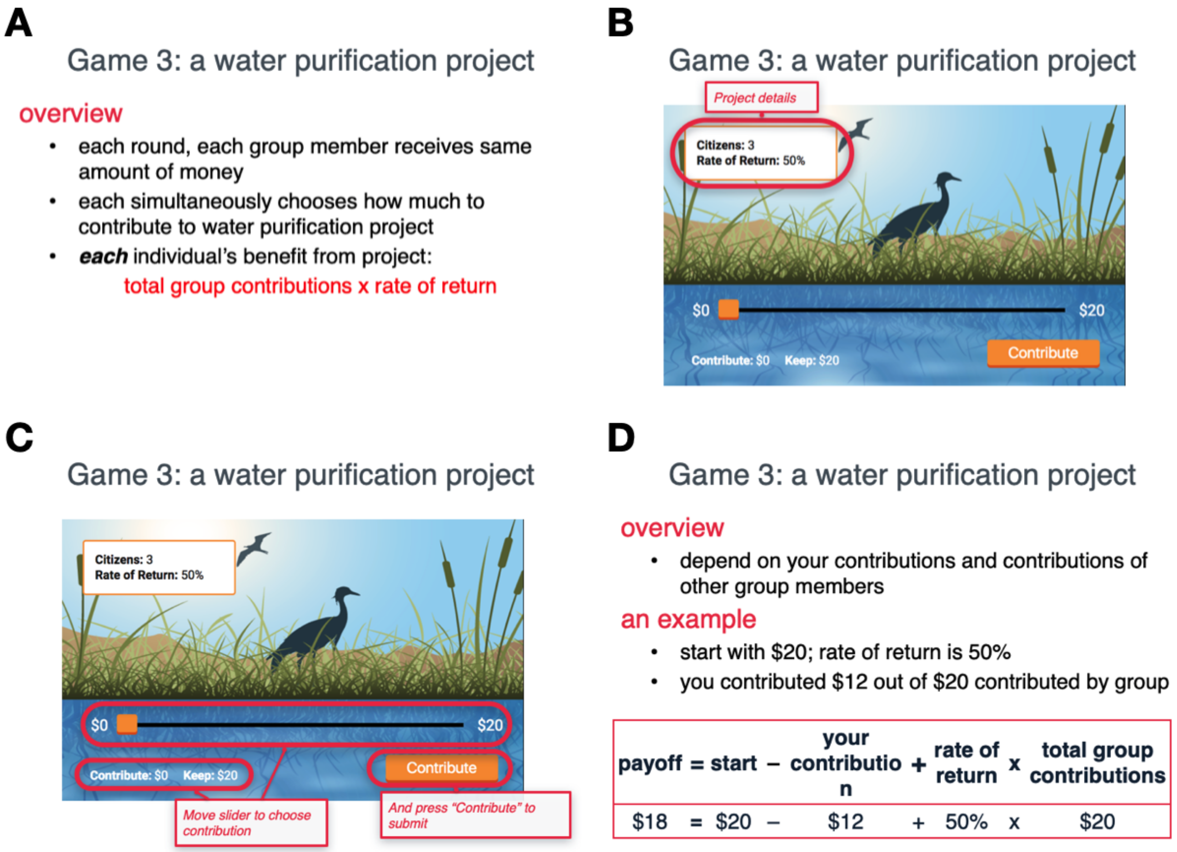

S21 Fig: Instruction slides and screenshots of the public good game. (A) Overview of the rules. (B) Player's initial screen. (C) shows that the decision can be made by moving the slider. (D) An example of payoff calculation.

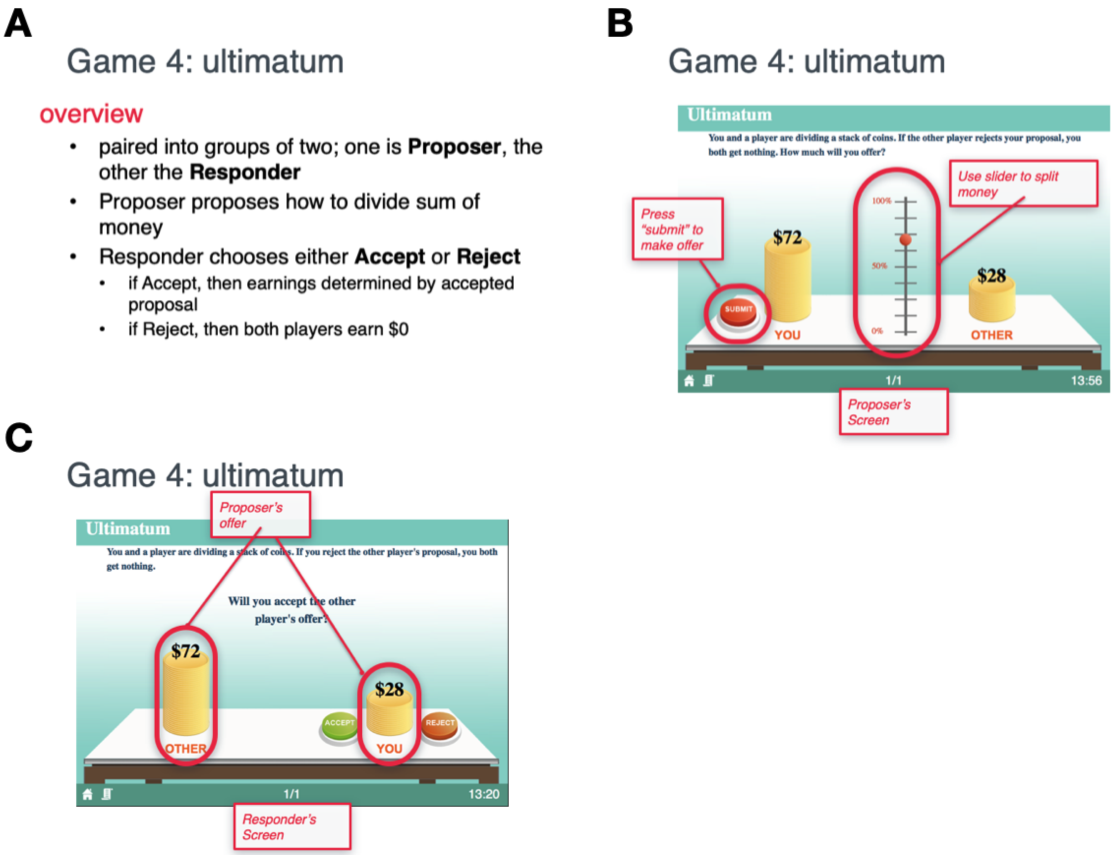

S22 Fig: Instruction slides and screenshots of the ultimatum game. (A) Overview of the rules. (B) Proposer's decision screen. (C) Responder's decision screen.

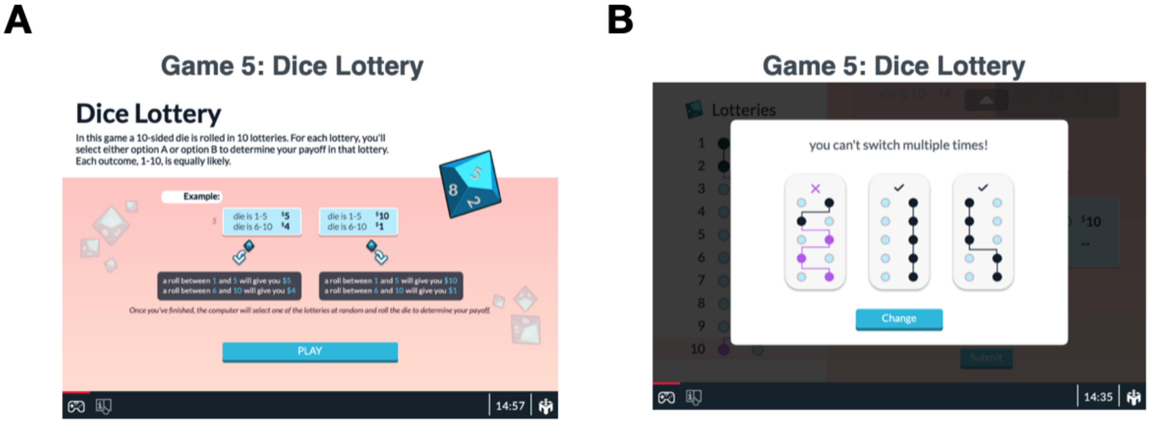

S23 Fig: Instruction slides and screenshots of the risk elicitation task. (A) Overview of the rules. (B) shows the restriction that the players can only switch at most once.

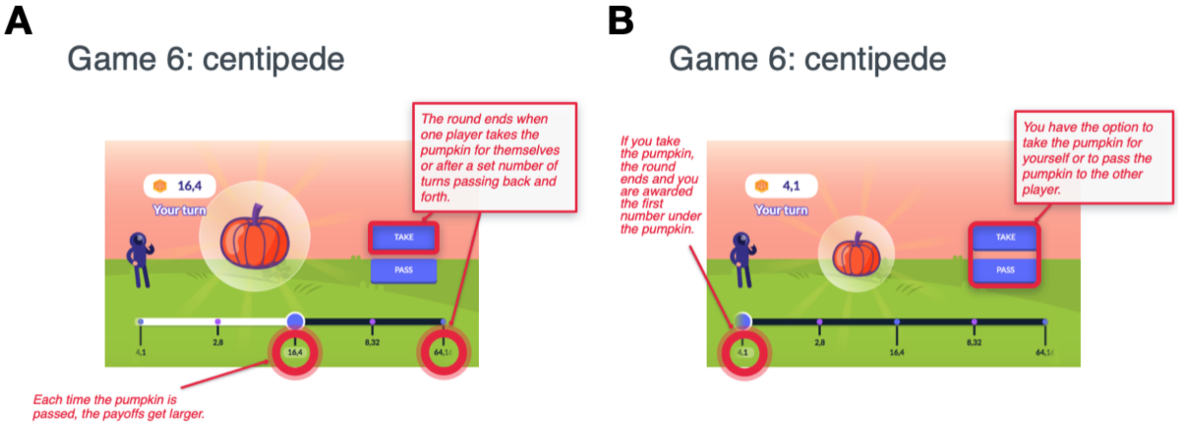

S24 Fig: Instruction slides and screenshots of the centipede game. (A) Overview of the game tree. (B) shows that the players can either "take" or "pass."

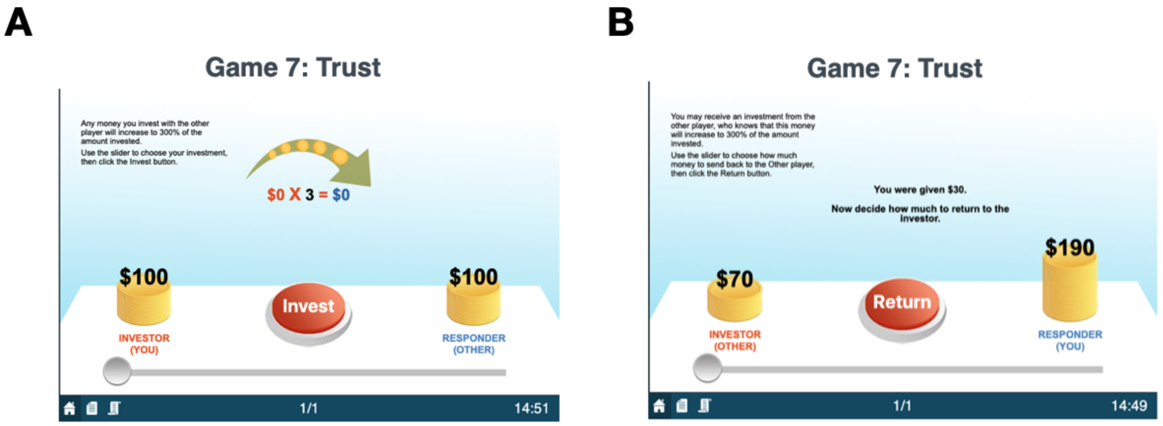

S25 Fig: Instruction slides and screenshots of the trust game. (A) Investor's decision screen. (B) Returner's decision screen.

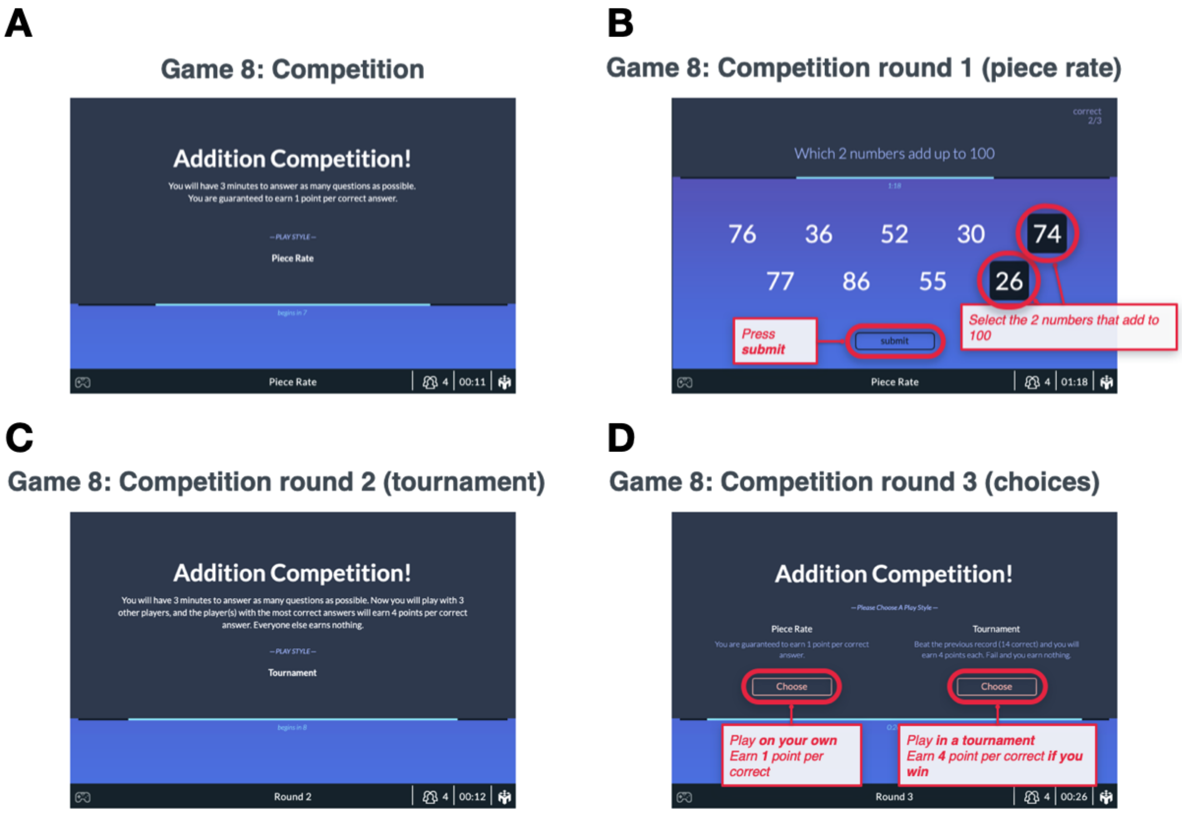

S26 Fig: Instruction slides and screenshots of the math competition game.(A) The initial screen of round one (piece rate). (B) Overview of the task. (C) The initial screen of round one (tournament). (D) Player's decision screen for the payoff scheme in round three.

E.3 Game Configurations

In this section, we provide the description of the game configurations in our experiment. Notice that we implement the same playlist in the same order in Experiment 1 and 2.

1. An N-player p-beauty contest game, where $p = 2/3$ and range is $[0, 100]$ played 3 rounds. $N = 100$ in group A and $N = 10$ in group B.

2. An N-player voter turnout game played 3 rounds. The party distribution (majority vs. minority) is 2 to 1. The payoff of winning the election is 100 and losing the election is 0. The cost of voting is uniformly distributed from 0 to 80. $N = 100$ in group A and $N = 10$ in group B.

3. An N-player linear public good game played for 8 rounds. The endowment is 20 units and MPCR is 30%. $N = 100$ in group A and $N = 10$ in group B.

4. A 2-player ultimatum bargaining game played once. The pie size is 100 units.

5. An individual, Holt-Laury risk elicitation task, played once. The configuration is shown in S22 Table. Notice that the decision maker can only switch between two options once.

S22 Table: Risk Elicitation Task

Option A	Option B	Expected Payoff Difference
1/10 of \$5, 9/10 of \$4	1/10 of \$10, 9/10 of \$1	\$2.2
2/10 of \$5, 8/10 of \$4	2/10 of \$10, 8/10 of \$1	\$1.4
3/10 of \$5, 7/10 of \$4	3/10 of \$10, 7/10 of \$1	\$0.6
4/10 of \$5, 6/10 of \$4	4/10 of \$10, 6/10 of \$1	-\$0.2
5/10 of \$5, 5/10 of \$4	5/10 of \$10, 5/10 of \$1	-\$1.0
6/10 of \$5, 4/10 of \$4	6/10 of \$10, 4/10 of \$1	-\$1.8
7/10 of \$5, 3/10 of \$4	7/10 of \$10, 3/10 of \$1	-\$2.6
8/10 of \$5, 2/10 of \$4	8/10 of \$10, 2/10 of \$1	-\$3.4
9/10 of \$5, 1/10 of \$4	9/10 of \$10, 1/10 of \$1	-\$4.2
10/10 of \$5, 0/10 of \$4	10/10 of \$10, 0/10 of \$1	-\$5.0

6. A 2-player centipede game, played 3 rounds. The game tree is shown in S27 Fig.

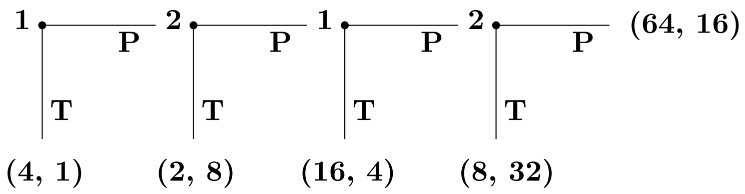

S27 Fig: The 2-player, 4 decision node centipede game. Payoffs in points to (Player 1, Player 2) are shown at the end of each terminal node.

7. A 2-player trust game, played once. Player 1 has endowment 100 units and player 2 has no endowment. The multiplier for player 1's investment is 3.

8. An individual, 3-stage real-effort task (called "math competition") exploring gender differences in compensation schemes. The compensation schemes for stage 1 and stage 2 are "piece rate" and "tournament," respectively. Before the third stage, the decision maker can decide either one to be the compensation scheme for the final stage.

References

Agranov, M., Potamites, E., Schotter, A., and Tergiman, C. (2012). Beliefs and endogenous cognitive levels: An experimental study. *Games and Economic Behavior*, 75(2):449–463.

Alaoui, L. and Penta, A. (2016). Endogenous depth of reasoning. *The Review of Economic Studies*, 83(4):1297–1333.

Andreoni, J. and Bernheim, B. D. (2009). Social image and the 50–50 norm: A theoretical and experimental analysis of audience effects. *Econometrica*, 77(5):1607–1636.

Berg, J., Dickhaut, J., and McCabe, K. (1995). Trust, reciprocity, and social history. *Games and Economic Behavior*, 10(1):122–142.

Camerer, C. F. (2003). *Behavioral Game Theory: Experiments in strategic interaction.* Princeton University Press.

Chabris, C. F., Morris, C. L., Taubinsky, D., Laibson, D., and Schuldt, J. P. (2009). The allocation of time in decision-making. *Journal of the European Economic Association*, 7(2-3):628–637.

Croson, R. and Gneezy, U. (2009). Gender differences in preferences. *Journal of Economic Literature*, 47:448–474.

Cubel, M. and Sanchez-Pages, S. (2017). Gender differences and stereotypes in strategic reasoning. *The Economic Journal*, 127:728–756.

Dean, M. and Ortoleva, P. (2019). The empirical relationship between nonstandard economic behaviors. *Proceedings of the National Academy of Sciences*, 116(33):16262–16267.

Filippin, A. and Crosetto, P. (2016). A reconsideration of gender differences in risk attitudes. *Management Science*, 62:3138–3160.

Güth, W., Schmittberger, R., and Schwarze, B. (1982). An experimental analysis of ultimatum bargaining. *Journal of Economic Behavior & Organization*, 3(4):367–388.

Holt, C. A. and Laury, S. K. (2002). Risk aversion and incentive effects. *American Economic Review*, 92(5):1644–1655.

Isaac, R. M., Walker, J. M., and Williams, A. W. (1994). Group size and the voluntary provision of public goods: Experimental evidence utilizing large groups. *Journal of Public Economics*, 54(1):1–36.

Konovalov, A. and Krajbich, I. (2019). Revealed strength of preference: Inference from response times. *Judgment & Decision Making*, 14(4).

Krajbich, I., Oud, B., and Fehr, E. (2014). Benefits of neuroeconomic modeling: new policy interventions and predictors of preference. *American Economic Review*, 104(5):501–06.

Lin, P.-H., Brown, A. L., Imai, T., Wang, J. T.-y., Wang, S. W., and Camerer, C. F. (2020). Evidence of general economic principles of bargaining and trade from 2,000 classroom experiments. *Nature Human Behaviour*, 4(9):917–927.

McKelvey, R. D. and Palfrey, T. R. (1992). An experimental study of the centipede game. *Econometrica*, pages 803–836.

Nagel, R. (1995). Unraveling in guessing games: An experimental study. *The American Economic Review*, 85(5):1313–1326.

Niederle, M. and Vesterlund, L. (2007). Do women shy away from competition? do men compete too much? *The Quarterly Journal of Economics*, 122(3):1067–1101.

Qin, B. (2020). Gender and the beauty contest game. *Working Paper*.

Roth, A. E., Prasnikar, V., Okuno-Fujiwara, M., and Zamir, S. (1991). Bargaining and market behavior in jerusalem, ljubljana, pittsburgh, and tokyo: An experimental study. *The American economic review*, pages 1068–1095.
