# Supplementary material for: Conducting large, repeated, multi-game economic experiments using mobile platforms
Source: PLoS One. 2021 Apr 29;16(4):e0250668. doi: 10.1371/journal.pone.0250668 (PMC8084158; doi:10.1371/journal.pone.0250668)
Supplement: Attachment — Submitted filename: PONE-D-20-38803 Report.pdf [file pone.0250668.s002.pdf]

Report on "Conducting Large, Repeated Multi-Game Economic Experiments Using Mobile Platforms" (PONE-D-20-38803)

Summary

This study reports on a set of experiments in which participants play eight classic laboratory games and decision-making tasks. The authors state three main contributions:

(1) Large-scale experimentation using mobile platforms, in-person or remotely, yields results that are consistent with previous findings from laboratory studies.

(2) The large number of subjects is used to explore how group size differences (10 subjects versus 100 subjects) affect people's play in the p-beauty contest game, a voter turnout game, and a linear public goods game. The authors find significant differences in behavior depending on group size.

(3) There are some interesting correlations in behavior *across* games and decision-making tasks (within-subject design). For example, the authors state that "the greater the strategic sophistication of a subject in the p-beauty contest game, the more likely that subject is to be a free rider in the public goods game or to offer very little as the first mover in the ultimatum game."

Comments

This is an interesting paper. The analysis is done well and the results are communicated in a succinct and informative way. The paper is a bit incomplete at times. The authors point to several interesting patterns in the data, but none of them is explored in terms of the implied economics/underlying mechanisms. Instead, to elevate the contribution of the paper, it is stated that the methodology of the paper is an important contribution, which I don't fully agree with. Below I offer some comments to explain in more detail what I mean.

(1) The introduction reviews the related literature. Because the experiment consists of eight different games, the authors have to focus on a narrow set of studies to cite and discuss. This is fine. However, after reading the introduction (and even after reading the entire paper), it remains unclear to me what specifically the current experiment adds to the literature. The authors correctly say that there are many studies that look at the effects of group size, also with very large groups, and that there are many studies that examine correlations in behavior across games. For example, with regard to the latter, the authors write that "most of the studies exploring correlations in strategic behavior across games involve only two games, whereas we look for interaction effects across eight different games or tasks that have been widely studied." I'm not sure that looking at eight games instead of two games is a good thing… if anything this seems to be a disadvantage because this is a within-subject experiment. Of course, there *is* an advantage of running so many games/tasks, which is that there is a larger potential to find interesting and new results. But then these new results should be the contribution of the paper, not the fact that eight games are analyzed. Similarly, it remains blurry what new insights are provided in terms of group size.

(2) Most of the reported results replicate results from the existing literature: in the beauty contest, larger group size results in behavior closer to equilibrium; in the turnout game, there is a missing underdog effect; in the linear public good game, group size has no clear effect on contributions. Such replication is of course very useful. However, the fact that these effects can be replicated does not necessarily mean that the used design/methodology is particularly desirable. Indeed, one would expect these results to be somewhat robust in most settings (e.g., classroom experiments, but this doesn't mean classroom experiments have no issues). Rather the fact that the key results replicate well would strengthen my confidence in potential novel results that are reported. One such interesting finding is that in the public goods game, there is "a greater number of extreme behavioral types in strong free-riders and altruists." I think it should be "*greater percentage"* instead of "*greater number"* here, in line with the appendix. But more importantly, is this finding new? Why does this happen? Wouldn't it mean that there might not be fixed "behavioral types" as the literature sometimes assumes, but rather people change their expectations depending on the size of the group and thus behave differently. There is an important question here: if in a public good game with, say, four players, the type distribution differs markedly from the one in the same game with more players, wouldn't this mean that the key thing we all should be studying is expectations formation (or types of expectation formation) rather than trying to classify "behavioral types" based on preferences?

(3) The results reported in the section "Behavior across games" have limited significance to me. Because the experiment was run as part of a summer camp, there are large selection effects. This is not necessarily a problem for testing specific hypotheses that should hold independent of the subject pool. But in this section, the authors report relationships between behaviors across games, based on *exploratory* data analysis. It remains unclear what empirical approach the authors used: Were there at least implicit hypotheses when analyzing the data across games? These should be explained. An exploratory analysis can of course be very useful, but much more so in a more representative samples, such as in the paper on "Econographics" that the authors cite. There is also the issue that subjects could be hedging across games, because they are told that their decisions and corresponding points earned from all games would matter for their final payment. Because of this, the results from this section probably have to be taken with a grain of salt.

(4) The authors write "we report on a break-through, incentivized experimental study involving more than 1,200 university students playing eight classic laboratory games or individual decision-making tasks." This sentence suggests that the sample size of 1,200 is a breakthrough… but there are many lab experiments with just as many (and more) subjects. Maybe the authors mean that the large-group treatments are a breakthrough for experimental economics, but even this is a bit much to state. Similarly, the authors suggest that group size effects are "overlooked" in the literature. This is not true given the many papers whose entire purpose is to vary the group size. What (I think) they mean to say is that some of the results we think might hold generally in the literature, might not be stable under changing group sizes. But which results exactly do they have in mind, i.e., do they think this holds beyond the games they study? At least for the studied games, I believe there was at least one study that has previously looked at group size? Anyway, the claim that group size is more important than we previously thought seems unfounded to me.

(5) The authors write that "Furthermore, Fig 5 shows that the more risk averse players are significantly less likely to be strong free riders...." Since this is a repeated game, and it is well-known that a finite repeated game does usually not correspond to the one-shot version (even if SPE would predict so), wouldn't it make sense for a risk-averse person to try to cooperate *initially*? Vice versa, a risk-loving person might free ride hoping others will still sustain cooperation. This reasoning may be wrong, but I believe the result should be explained better. Similarly, the result that investment behavior in the trust game is potentially related to the belief forming ability in a multi-stage game rather than risk preferences is really quite intriguing, but again without a conceptual framework or at least a deeper analysis of this effect, the result is uninterpretable. This is what I meant above when I said the discussion of the results is incomplete.

(6) Line 116: "This finding is rather intuitive: in larger groups, subjects are primed by the greater competitive pressure to iterate their reasoning further than they would in smaller, less competitive groups." I do not fully understand this observation. In a large group, is it not the case that a subject is very unlikely to "win" and hence may be inclined to exert less effort and do less iterations in the beauty contest game rather than more? Again, a clearer discussion would be useful. The fact that the paper is brief is great, but I believe currently, at the key moments when an interesting economic result pops up, it is not explored and explained in sufficient detail.

(7) Conclusion line 313: "At the same time, we are able to leverage our large-scale, multi-game approach to obtain interesting new findings on group size effects and correlations in strategic behavior across games that would be difficult to obtain in traditional, limited capacity laboratory settings." I really think that this should be *the* contribution of the paper. The other claims about a novel way and proof-of-concept for conducting large-scale experiments in a post-pandemic world, at least to me, feels a bit like reinventing the wheel. If this were true, this paper should be published in Nature/Science. Instead, I believe it would be beneficial, in addition to the replication results, to further develop the new findings the paper provides. For example, one could discuss mechanisms why the percentage of pure free-riders/altruists increases with the group size in a public good game.

Overall, the experiment is interesting, because it features such large groups in some cases. The results are reported appropriately in a statistical sense, but there is almost no interpretation/development of the findings, and it is also not clear which of the findings are truly new.
