# Supplementary material for: Conducting large, repeated, multi-game economic experiments using mobile platforms
Source: PLoS One. 2021 Apr 29;16(4):e0250668. doi: 10.1371/journal.pone.0250668 (PMC8084158; doi:10.1371/journal.pone.0250668)
Supplement: Attachment — Submitted filename: Report.pdf [file pone.0250668.s003.pdf]

Referee Report on "Conducting Large, Repeated Multi-Game Economic Experiments Using Mobile Platforms"

Summary

This paper reports the results of two large economic experiments conducted on mobile platforms. The first experiment was conducted in-person, while subjects participated remotely in the second experiment. Both experiments included economic games and tasks that have been studied through lab experiments in the past. The paper studies how behavior in three games varies with group size and how individual choices are correlated across different games and tasks.

Evaluation

I think this is an interesting paper. The paper claims to make the following contributions: 1) providing a proof-of-concept that large economics experiments can be conducted on mobile platforms, 2) exploiting the large participant pool to study the effect of large group sizes on behavior in games, 3) studying the correlation between choices and behaviors across different tasks. While the third contribution feels a bit disconnected from the others, I think the first two contributions are potentially very important. I think it is reasonable to assume that we will see a continuous shift away from the traditional lab and towards mobile platforms in experimental economics. Against this backdrop, this paper can provide useful evidence on the robustness of experimental methods and results to new mobile-based protocols. It also suggests ways in which the new technologies can be fruitfully employed for research in experimental economics, namely leveraging the large number of participants available in online studies.

My main concern is that to actually provide a useful reference for future mobile-based studies, this paper should provide a clearer comparison with standard lab experiments. The paper repeatedly argues that the experiments replicate several existing findings for small groups (see the abstract for example), but I do not think the paper actually shows this. For each of the three games, the paper comments on whether the effect of group size is consistent with estimates previously obtained in other studies. However, the paper does not discuss whether the results from the small groups treatment are similar or different from those of traditional lab studies. So, I would like the paper to provide a better analysis of whether the experiments replicate existing findings for small groups, for each of the three games and the other tasks as well. The comparison with previous lab studies should be done separately for experiment 1 and experiment 2. This can give the readers an idea of how the results are affected by whether the experiment is run in the traditional lab setting vs. in-person on mobile platforms vs. remotely on mobile platforms. I do not require a fully quantitative comparison (i.e. formal hypothesis testing). Below I describe exactly which points should be addressed for each game in the experiment.

1. Beauty contest game
    a. How does the distribution of guesses observed in the small-group experiment compare to that in previous lab experiments with a similar parametrization?

b. How do the changes in average guesses across rounds compare to the dynamics of average guesses in previous lab experiments?

2. Voter turnout game

a. The paper finds that turnout is generally much greater than predicted by theory. Is this consistent with previous findings from lab experiments?

b. How do turnout rates observed in the small-group experiment (across the majority and minority teams) compare to those in previous lab experiments with a similar parametrization?

3. Public good game

a. How does the average contribution observed in the small-group experiment compare to that in previous lab experiments with a similar parametrization?

b. How does the distribution of player types in the small-group experiment compare to that in previous lab experiments?

c. How do the changes in the average contribution across rounds compare to the dynamics of average contributions in previous lab experiments?

4. Other games and tasks

I think it would be useful to highlight whether the results of the other games in the experiments replicate previous findings from lab experiments. Currently the results of the other games are presented in the supplementary materials. I have also noticed that, for most of these games, the supplementary materials already include a comparison with previous findings. I think these comparisons should be included in the main body of the paper. Note that I am not recommending to move the complete analysis of results in the main body, but I would like the paper to briefly highlight to what extend the remaining games in experiment 1 and experiment 2 replicate previous findings.

5. If after making the revisions suggested above the length of the paper exceeds the limit, I recommend shortening the section on “Behavior across games.” While this section is interesting, studying correlations across games feels a bit disconnected from the main contributions of the paper (which are comparing mobile-based experiments to traditional lab experiments and exploiting the large subject pool of mobile-based experiments).
