# Supplementary material for: Conducting large, repeated, multi-game economic experiments using mobile platforms
Source: PLoS One. 2021 Apr 29;16(4):e0250668. doi: 10.1371/journal.pone.0250668 (PMC8084158; doi:10.1371/journal.pone.0250668)
Supplement: Attachment — Submitted filename: plos_one_response_letter.pdf [file pone.0250668.s004.pdf]

Response to Reviewers on "Conducting Large, Repeated Multi-Game Economic Experiments Using Mobile Platforms" (PONE-D-20-38803)

We thank the editor and the three referees for their comments and suggestions on our initial draft. In this response we indicate where and how we have responded to the editor's and referees' comments in the revised draft of our paper. For convenience, we repeat the comments we received and provide our response in boldface text.

Editor, Douglas Davis

...all (3 referees) like the manuscript, but also all suggest that the paper could be made considerably better with attention to several issues. Prominent concerns include a call for some clarification of the paper's specific methodological contribution, a more careful comparison of results from your large sample remote sessions with smaller scale laboratory experiments, and more discussion of some of the potential costs of increasing the number of participants (such as payoff scalability).

We now clarify the methodological contributions of our paper in the introduction. Further, we now explicitly compare our experimental findings with studies that are the closest in design/parameterization/group sizes to our experimental implementations. Finally, we have included some discussion surrounding payoff scalability in moving from small to large size groups, for the beauty contest game where it is invariant and for the public goods game where it is not.

The readers also all seem to take issue with your section comparing performance across games.

Since only one of the referees liked our cross-game analysis, we have decided to put this discussion in the supplementary material.

At base, the readers all regard large scale remote experiments as an important possible direction for the future of experimental economics. The common thread of their comments is that they would like a more thorough consideration of what this alternative approach may offer, when it may be useful and what costs it may impose.

We have endeavored to address all of these comments in the revised draft as detailed in the individual responses to the referees below. We now provide a marked-up copy of our manuscript that highlights changes made to the original version.

Journal Requirements:

When submitting your revision, we need you to address these additional requirements.

1. Please ensure that your manuscript meets PLOS ONE's style requirements, including those for file naming. The PLOS ONE style templates can be found at https://journals.plos.org/plosone/s/file?id=wjVg/PLOSOne_formatting_sample_main_body.pdf and https://journals.plos.org/plosone/s/file?id=ba62/PLOSOne_formatting_sample_title_authors_affiliations.pdf

- **Response: We have set the manuscript into PLOS ONE template and modified the file names to comply with PLOS ONE requirements.**

2. We note that the grant information you provided in the 'Funding Information' and 'Financial Disclosure' sections do not match. When you resubmit, please ensure that you provide the correct grant numbers for the awards you received for your study in the 'Funding Information' section.

- **Response: We have updated the financial disclosure so that it matches with the funding information in the main text.**

3. Thank you for stating the following in the Financial Disclosure section: "Funding: Z.L. was supported by National Natural Science Foundation of China (Grant No. 71873116) and the NSFC Basic Science Center Program (Grant No. 71988101). Experiment 1 was funded by MobLab. Experiment 2 was funded by Xiamen University." We note that one or more of the authors have an affiliation to the commercial funders of this research study: MobLab Inc.

   3.1. Please provide an amended Funding Statement declaring this commercial affiliation, as well as a statement regarding the Role of Funders in your study. If the funding organization did not play a role in the study design, data collection and analysis, decision to publish, or preparation of the manuscript and only provided financial support in the form of authors' salaries and/or research materials, please review your statements relating to the author contributions, and ensure you have specifically and accurately indicated the role(s) that these authors had in your study. You can update author roles in the Author Contributions section of the online submission form. Please also include the following statement within your amended Funding Statement. "The funder provided support in the form of salaries for authors [insert relevant initials] but did not have any additional role in the study design, data collection and analysis, decision to publish, or preparation of the manuscript. The specific roles of these authors are articulated in the 'author contributions' section." If your commercial affiliation did play a role in your study, please state and explain this role within your updated Funding Statement.

   3.2. Please also provide an updated Competing Interests Statement declaring this commercial affiliation along with any other relevant declarations relating to employment, consultancy, patents, products in development, or marketed products, etc. Within your Competing Interests Statement, please confirm that this commercial affiliation does not alter your adherence to all PLOS ONE policies on sharing data and materials by including the following statement: "This does not alter our adherence to PLOS ONE policies on sharing data and materials." (as detailed online in our guide for authors http://journals.plos.org/plosone/s/competing-interests). If this adherence statement is not accurate and there are restrictions on sharing of data and/or materials, please state these. Please note that we cannot proceed with consideration of your article until this information has been declared.

   Please include both an updated Funding Statement and Competing Interests Statement in your cover letter. We will change the online submission form on your behalf.

   Please know it is PLOS ONE policy for corresponding authors to declare, on behalf of all authors, all potential competing interests for the purposes of transparency. PLOS defines a competing interest as anything that interferes with, or could reasonably be perceived as interfering with, the full and objective presentation, peer review, editorial decision-making, or publication of research or non-research articles submitted to one of the journals. Competing interests can be financial or non-financial, professional, or personal. Competing interests can arise in relationship to an organization or another person. Please follow this link to our website for more details on competing interests: http://journals.plos.org/plosone/s/competing-interests

- **Response: We have included the additional statements in Funding Statement and Competing Interests Statements.**

4. We note that you have stated that you will provide repository information for your data at acceptance. Should your manuscript be accepted for publication, we will hold it until you provide the relevant accession numbers or DOIs necessary to access your data. If you wish to make changes to your Data Availability statement, please describe these changes in your cover letter and we will update your Data Availability statement to reflect the information you provide.

- **Response: The experimental data and code for all the analyses can be found on the Open Science Framework. The link is attached in the Data Availability statement, and is currently for peer review only. We will lift the embargo once the manuscript is accepted.**

5. PLOS requires an ORCID iD for the corresponding author in Editorial Manager on papers submitted after December 6th, 2016. Please ensure that you have an ORCID iD and that it is validated in Editorial Manager. To do this, go to 'Update my Information' (in the upper left-hand corner of the main menu), and click on the Fetch/Validate link next to the ORCID field. This will take you to the ORCID site and allow you to create a new iD or authenticate a pre-existing iD in Editorial Manager. Please see the following video for instructions on linking an ORCID iD to your Editorial Manager account: https://www.youtube.com/watch?v=_xcclfuvtxQ

- **Response: The ORCID id for the corresponding author has been updated in the system.**

6. We note that Figure S1 and S2 in your submission contain map images which may be copyrighted. All PLOS content is published under the Creative Commons Attribution License (CC BY 4.0), which means that the manuscript, images, and Supporting Information files will be freely available online, and any third party is permitted to access, download, copy, distribute, and use these materials in any way, even commercially, with proper attribution. For these reasons, we cannot publish previously copyrighted maps or satellite images created using proprietary data, such as Google software (Google Maps, Street View, and Earth). For more information, see our copyright guidelines: http://journals.plos.org/plosone/s/licenses-and-copyright. We require you to either (1) present written permission from the copyright holder to publish these figures specifically under the CC BY 4.0 license, or (2) remove the figures from your submission:

    6.1. You may seek permission from the original copyright holder of Figure S1 and S2 to publish the content specifically under the CC BY 4.0 license. We recommend that you contact the original copyright holder with the Content Permission Form (http://journals.plos.org/plosone/s/file?id=7c09/content-permission-form.pdf) and the following text: "I request permission for the open-access journal PLOS ONE to publish XXX under the Creative Commons Attribution License (CCAL) CC BY 4.0 (http://creativecommons.org/licenses/by/4.0/). Please be aware that this license allows unrestricted use and distribution, even commercially, by third parties. Please reply and provide explicit written permission to publish XXX under a CC BY license and complete the attached form." Please upload the completed Content Permission Form or other proof of granted permissions as an "Other" file with your submission. In the figure caption of the copyrighted figure, please include the following text: "Reprinted from [ref] under a CC BY license, with permission from [name of publisher], original copyright [original copyright year]."

    6.2. If you are unable to obtain permission from the original copyright holder to publish these figures under the CC BY 4.0 license or if the copyright holder's requirements are incompatible with the CC BY 4.0 license, please either i) remove the figure or ii) supply a replacement figure that complies with the CC BY 4.0 license. Please check copyright information on all replacement figures and update the figure caption with source information. If applicable, please specify in the figure caption text when a figure is similar but not identical to the original image and is therefore for illustrative purposes only. The following resources for replacing copyrighted map figures may be helpful:

        * USGS National Map Viewer (public domain): http://viewer.nationalmap.gov/viewer/
        * The Gateway to Astronaut Photography of Earth (public domain): http://eol.jsc.nasa.gov/sseop/clickmap/
        * Maps at the CIA (public domain): https://www.cia.gov/library/publications/the-world-factbook/index.html and https://www.cia.gov/library/publications/cia-maps-publications/index.html
        * NASA Earth Observatory (public domain): http://earthobservatory.nasa.gov/
        * Landsat: http://landsat.visibleearth.nasa.gov/

    * USGS EROS (Earth Resources Observatory and Science (EROS) Center) (public domain): http://eros.usgs.gov/#
    * Natural Earth (public domain): http://www.naturalearthdata.com/

- **Response: We have removed Figure S1 and S2 from the supplementary information.**

Reviewer 1

This is an interesting paper. The analysis is done well and the results are communicated in a succinct and informative way. The paper is a bit incomplete at times. The authors point to several interesting patterns in the data, but none of them is explored in terms of the implied economics/underlying mechanisms. Instead, to elevate the contribution of the paper, it is stated that the methodology of the paper is an important contribution, which I don't fully agree with. Below I offer some comments to explain in more detail what I mean.

1. The introduction reviews the related literature. Because the experiment consists of eight different games, the authors have to focus on a narrow set of studies to cite and discuss. This is fine. However, after reading the introduction (and even after reading the entire paper), it remains unclear to me what specifically the current experiment adds to the literature. The authors correctly say that there are many studies that look at the effects of group size, also with very large groups, and that there are many studies that examine correlations in behavior across games. For example, with regard to the latter, the authors write that "most of the studies exploring correlations in strategic behavior across games involve only two games, whereas we look for interaction effects across eight different games or tasks that have been widely studied." I'm not sure that looking at eight games instead of two games is a good thing... if anything this seems to be a disadvantage because this is a within subject experiment. Of course, there is an advantage of running so many games/tasks, which is that there is a larger potential to find interesting and new results. But then these new results should be the contribution of the paper, not the fact that eight games are analyzed. Similarly, it remains blurry what new insights are provided in terms of group size.

- **Response: In response to your comments we have tried to clarify the contributions of the paper in the revised introduction. Further, we have moved the discussion of behavior across games to the supplementary material. Finally, we have gone into greater detail as to how our group size effects relate to the literature, with the aim of making these results less blurry.**

2. Most of the reported results replicate results from the existing literature: in the beauty contest, larger group size results in behavior closer to equilibrium; in the turnout game, there is a missing underdog effect; in the linear public good game, group size has no clear effect on contributions. Such replication is of course very useful. However, the fact that these effects can be replicated does not necessarily mean that the used design/methodology is particularly desirable. Indeed, one would expect these results to be somewhat robust in most settings (e.g., classroom experiments, but this doesn't mean classroom experiments have no issues). Rather the fact that the key results replicate well would strengthen my confidence in potential novel results that are reported. One such interesting finding is that in the public goods game, there is "a greater number of extreme behavioral types in strong free-riders and altruists." I think it should be "greater percentage" instead of "greater number" here, in line with the appendix. But more importantly, is this finding new? Why does this happen? Wouldn't it mean that there might not be fixed "behavioral types" as the literature sometimes assumes, but rather people change their expectations depending on the size of the group and thus behave differently. There is an important question here: if in a public good game with, say, four players, the type distribution differs markedly from the one in the same game with more players, wouldn't this mean that the key thing we all should be studying is expectations formation (or types of expectation formation) rather than trying to classify "behavioral types" based on preferences?

- **Response: Thank you for these comments. We agree that replication is important. We now use "proportion" rather than "number". Yes, we are not aware of any prior work looking at the constancy in the proportions of behavioral types across public good games with different group sizes, so this is a new finding. Commenting on this finding, we write, "These differences in the proportions of player types by group size are new and suggest that individual behavior may be quite malleable and dependent on the size of the group that subjects find themselves in. For instance, subjects in the large (small) group treatment might believe that free-riding is more likely (less likely) and might respond to such**

beliefs by contributing more (less). Future work on this topic would require a more careful, within-subject experimental design that varied the group size and elicited subjects' beliefs about the contributions of others."

3. The results reported in the section "Behavior across games" have limited significance to me. Because the experiment was run as part of a summer camp, there are large selection effects. This is not necessarily a problem for testing specific hypotheses that should hold independent of the subject pool. But in this section, the authors report relationships between behaviors across games, based on exploratory data analysis. It remains unclear what empirical approach the authors used: Were there at least implicit hypotheses when analyzing the data across games? These should be explained. An exploratory analysis can of course be very useful, but much more so in a more representative samples, such as in the paper on "Econographics" that the authors cite. There is also the issue that subjects could be hedging across games, because they are told that their decisions and corresponding points earned from all games would matter for their final payment. Because of this, the results from this section probably have to be taken with a grain of salt.

- **Response: Thank you for this comment. We have moved the discussion of behavior across games to the supplementary material. In the main text we now write only that "The collection of data on the play of many games by the *same* subjects enables comparisons to be made in the way subjects behave across games, which we believe will be an important area for future research using the large scale mobile experimental methodology that we propose here, and we provide some preliminary analysis in the supplemental material."**

4. The authors write "we report on a break-through, incentivized experimental study involving more than 1,200 university students playing eight classic laboratory games or individual decision making tasks." This sentence suggests that the sample size of 1,200 is a breakthrough... but there are many lab experiments with just as many (and more) subjects. Maybe the authors mean that the large-group treatments are a breakthrough for experimental economics, but even this is a bit much to state. Similarly, the authors suggest that group size effects are "overlooked" in the literature. This is not true given the many papers whose entire purpose is to vary the group size. What (I think) they mean to say is that some of the results we think might hold generally in the literature, might not be stable under changing group sizes. But which results exactly do they have in mind, i.e., do they think this holds beyond the games they study? At least for the studied games, I believe there was at least one study that has previously looked at group size? Anyway, the claim that group size is more important than we previously thought seems unfounded to me.

- **Response: We are now more clear about what we regard our break-through to be. It is not the number of subjects, as indeed there are other studies with similar numbers of subjects to ours. Rather, as we now write in the introduction, "we report on a break-through, incentivized experimental study comparing the behavior of subjects playing eight classic interactive laboratory games or individual decision-making tasks either in-person or remotely using entirely mobile devices." So our view of the breakthrough is, using subjects own mobile devices we compare behavior in person versus remotely. We also argue that our approach blurs the lines between lab and field experiments (or, if one prefers, "lab in the field" experiments).**

5. The authors write that "Furthermore, Fig 5 shows that the more risk averse players are significantly less likely to be strong free riders...." Since this is a repeated game, and it is well-known that a finite repeated game does usually not correspond to the one-shot version (even if SPE would predict so), wouldn't it make sense for a risk-averse person to try to cooperate initially? Vice versa, a risk-loving person might free ride hoping others will still sustain cooperation. This reasoning may be wrong, but I believe the result should be explained better. Similarly, the result that investment behavior in the trust game is potentially related to the belief forming ability in a multi-stage game rather than risk preferences is really quite intriguing, but again without a conceptual framework or at least a deeper analysis of this effect, the result is uninterpretable. This is what I meant above when I said the discussion of the results is incomplete.

- **Response: As noted earlier in response to your comment # 3, we no longer emphasize the comparison across games as an important contribution of our paper. We agree that some conceptual framework or a deeper analysis of the correlations we find would be required to interpret these findings. Still, we think theres is promise of our method for evaluating such a conceptual framework in future research.**

6. Line 116: "This finding is rather intuitive: in larger groups, subjects are primed by the greater competitive pressure to iterate their reasoning further than they would in smaller, less competitive groups." I do not fully understand this observation. In a large group, is it not the case that a subject is very unlikely to "win" and hence may be inclined to exert less effort and do less iterations in the beauty contest game rather than more? Again, a clearer discussion would be useful. The fact that the paper is brief is great, but I believe currently, at the key moments when an interesting economic result pops up, it is not explored and explained in sufficient detail.

- **Response: We have deleted the "intuitive phrasing" and now offer a second explanation for why group size should matter. Specifically, we now write: "We speculate that in larger groups, subjects may react to the greater competitive pressure by iterating their reasoning somewhat further than they would in smaller, less competitive groups. Alternatively, in larger groups the effects of outlier choices such as a guess of 100 may be more greatly diminished, and subjects may react to this difference by making guesses that are closer to the equilibrium prediction." We agree with you that one reaction subjects might have to being in a larger group is that they become less willing to engage in any reasoning at all, but the evidence seems to be strongly against this conjecture, as is now made even clearer by the comparison we now make with Thaler's (1997) data.**

7. Conclusion line 313: "At the same time, we are able to leverage our large-scale, multi-game approach to obtain interesting new findings on group size effects and correlations in strategic behavior across games that would be difficult to obtain in traditional, limited capacity laboratory settings." I really think that this should be the contribution of the paper. The other claims about a novel way and proof-of-concept for conducting large-scale experiments in a post-pandemic world, at least to me, feels a bit like reinventing the wheel. If this were true, this paper should be published in Nature/Science. Instead, I believe it would be beneficial, in addition to the replication results, to further develop the new findings the paper provides. For example, one could discuss mechanisms why the percentage of pure free-riders/altruists increases with the group size in a public good game.

   Overall, the experiment is interesting, because it features such large groups in some cases. The results are reported appropriately in a statistical sense, but there is almost no interpretation/development of the findings, and it is also not clear which of the findings are truly new.

- **Response: We now emphasize that group size differences are an important contribution of our paper and an innovation made possible by our new method of mobile data collection and payment. We have eliminated references to a "post-pandemic world." We now do a better job of interpreting/developing our findings and making it clear what is new and what replicates existing results. We provide one mechanism by which the percentage of pure free-riders/altruists might rationally vary with the group size in a public good game. Specifically, we now write: "subjects in the large (small) group treatment might believe that free-riding is more likely (less likely) and might respond to such beliefs by contributing more (less). Future work on this topic would require a more careful, within-subject experimental design that varied the group size and elicited subjects' beliefs about the contributions of others."**

Reviewer 2

I think this is an interesting paper. The paper claims to make the following contributions: 1) providing a proof-of-concept that large economics experiments can be conducted on mobile platforms, 2) exploiting the large participant pool to study the effect of large group sizes on behavior in games, 3) studying the correlation between choices and behaviors across different tasks. While the third contribution feels a bit disconnected from the others, I think the first two contributions are potentially very important. I think it is reasonable to assume that we will see a continuous shift away from the traditional lab and towards mobile platforms in experimental economics. Against this backdrop, this paper can provide useful evidence on the robustness of experimental methods and results to new mobile-based protocols. It also suggests ways in which the new technologies can be fruitfully employed for research in experimental economics, namely leveraging the large number of participants available in online studies.

- **Response: Thank you. We now emphasize the first two contributions in the introduction and we have relegated the correlation in behavior across tasks to the supplementary material.**

My main concern is that to actually provide a useful reference for future mobile-based studies, this paper should provide a clearer comparison with standard lab experiments. The paper repeatedly argues that the experiments replicate several existing findings for small groups (see the abstract for example), but I do not think the paper actually shows this. For each of the three games, the paper comments on whether the effect of group size is consistent with estimates previously obtained in other studies. However, the paper does not discuss whether the results from the small groups treatment are similar or different from those of traditional lab studies. So, I would like the paper to provide a better analysis of whether the experiments replicate existing findings for small groups, for each of the three games and the other tasks as well. The comparison with previous lab studies should be done separately for experiment 1 and experiment 2. This can give the readers an idea of how the results are affected by whether the experiment is run in the traditional lab setting vs. in-person on mobile platforms vs. remotely on mobile platforms. I do not require a fully quantitative comparison (i.e. formal hypothesis testing). Below I describe exactly which points should be addressed for each game in the experiment.

- **Response: We have found the experiments that most closely resemble our small size treatments for tasks 1-3 (e.g. in terms of parameterization and group size) and we now make explicit comparisons with these studies for both Experiment 1 and Experiment 2 in Figures 1-3.**

1. Beauty contest game
   a. How does the distribution of guesses observed in the small-group experiment compare to that in previous lab experiments with a similar parametrization?
   b. How do the changes in average guesses across rounds compare to the dynamics of average guesses in previous lab experiments?

- **Response: [a.] See the revised Figure 1. We now compare our results with Nagel's (1995) data for the treatment with $2/3\times$ the average and groups of size 15-18 over playing over several rounds. We find some differences in the distribution of guesses particularly between Nagel's data and our small group treatment but not between Nagel's data and our large group treatment. We speculate that Nagel's larger group sizes (5-8 more subjects) may play a role here, though we emphasize that more research would be needed to reach such a conclusion. In addition, we compare the distribution of first round guesses with Thaler's (1997) data from the one-shot Financial Times $2/3\times$*the average* beauty contest game with $N = 1468$. There we find a very large difference. In particular, the distribution of guesses in all our treatments/experiments (and Nagel's data as well) first-order stochastically dominate Thaler's (1997) data. Based on these differences we argue that the size of the group is important and deserving of more research. [b.] We find that for the first round, except for the large group treatment in**

Experiment 1, there is no difference between the mean guesses in Nagel's data and any treatment/experiment of our own data. In rounds 2-3 of experiment 1, the mean guesses in our small group are significantly higher than in Nagel's data, while for experiment 2 this is not the case. Mean guesses in rounds 2-3 of our large group treatment (both experiments 1 and 2) are not different from means guesses in rounds 2-3 of Nagel's data.

2. Voter turnout game

   a. The paper finds that turnout is generally much greater than predicted by theory. Is this consistent with previous findings from lab experiments?

   b. How do turnout rates observed in the small-group experiment (across the majority and minority teams) compare to those in previous lab experiments with a similar parametrization?

- **Response: [a.] Similar to our findings, turnout is generally higher than predicted in experimental tests of this game. We now provide a number of references. [b.] In the Revised Figure 2, we now compare our results with the most similar parameterization to our own experiment, Levine and Palfrey's (2007) experimental treatment with a ratio of majority to minority team members of 2:1 and groups of size 9 (small) and 51 (large). We find that Levine and Palfrey's data are closer to Bayesian Nash equilibrium predictions than our data. We attribute these differences to the greater instruction that LP gave subjects in their experiment - a comprehension quiz and two practice rounds, that was not feasible in our design.**

3. Public good game

   a. How does the average contribution observed in the small-group experiment compare to that in previous lab experiments with a similar parameterization?

   b. How does the distribution of player types in the small-group experiment compare to that in previous lab experiments?

   c. How do the changes in the average contribution across rounds compare to the dynamics of average contributions in previous lab experiments?

- **Response: The revised Figure 3 now addresses all of your questions. We compare our experimental data with that of the closest other study, Isaac et al. [1994] (IWW) who also examined groups of size $N = 10$ and $N = 100$ both with an MPCR of 0.3. [a.] The average small group contribution as a percentage of endowment in our experiments is not different from what IWW found over all rounds and over most of the individual rounds of the game. [b.] The distributions of free riders and altruists in our experiment are similar to what we found using IWW's data; there are more free riders in small groups and more altruists in large groups, though the differences are more significantly pronounced in IWW's data. [c.] The new Figure 3 reveals that changes across rounds in our small and large group treatments are very similar and not different from what IWW found in their small group treatment. We note that one possible explanation for why we do not observe the same large versus small group differences as in IWW is that IWW's large group treatment was not incentivized using money payments (as we do in our experiment); rather subjects in IWW were paid in extra course credit points.**

4. Other games and tasks

   – I think it would be useful to highlight whether the results of the other games in the experiments replicate previous findings from lab experiments. Currently the results of the other games are presented in the supplementary materials. I have also noticed that, for most of these games, the supplementary materials already include a comparison with previous findings. I think these comparisons should be included in the main body of the paper. Note that I am not recommending to move the complete analysis of results in the main body, but I would like the paper to briefly highlight to what extend the remaining games in experiment 1 and experiment 2 replicate previous findings.

- **Response: We have now done this.**

5. If after making the revisions suggested above the length of the paper exceeds the limit, I recommend shortening the section on "Behavior across games." While this section is interesting, studying correlations across games feels a bit disconnected from the main contributions of the paper (which are comparing mobile-based experiments to traditional lab experiments and exploiting the large subject pool of mobile-based experiments).

- **Response: We have relegated the section "behavior across games" to the supplementary material.**

Reviewer 3

The authors conduct a series of large incentivized experiments using a mobile platform (MobLab) in China. The experiments show that such large experiments are feasible. Some economic intuitions build on the intuition that single agents are unlikely to be consequential. The current research suggests some of these intuitions might be tested using online platforms.

1. The paper is a nice illustration of what the future of experimental economics might look like. Several authors (e.g. Snowberg, Taubinsky, etc.) are embedding incentivized experiments in representative surveys. The paper shows that it might be possible to move from individual decision making to interactive decision making. This is a qualitative leap. The authors illustrate that there might be some solutions around the corner. Of course, this should not be surprising for any person vaguely familiar with the gaming industry. The contribution of the paper is to show that large experiments can be conducted with samples familiar to experimentalist (college students). This is important because it allows to compare results from standard lab sessions with virtual sessions (albeit in the experiments reported in the paper, subjects seem to be 'captured' subjects). The paper should emphasize more the fact that the only thing they are varying is session size.

- **Response: We have now included references to the work of Snowberg and Taubinsky in the introduction. We now emphasize that for the first three games, the main treatment variable is the group size.**

2. An important concern with lab experiments is the nuisance costs associated with registering for an experiment, participating in an experiment, getting paid, etc. There is always a concern that those showing-up in an experiment are different from the population at large. The recent work by Snowberg and Yaariv (Testing the Waters: Behavior across Participant Pools) reiterates the fact that behavior is similar across subject pools. This paper adds to that evidence, but it provides a different kind of robustness test. A discussion of these alternative robustness test would be useful.

- **Response: We now discuss in the Introduction how our study complements Snowberg and Yariv in considering whether observer effects matter or not. The main difference, as we now mention, is that Snowberg and Yariv primariliy collect data on individual characteristics, e.g. risk-taking, cognitive abilities, confidence, etc., while we primarily collect data on interactive behavior in games. Further, we use subjects own mobile devices in both our in-person and remote comparisons.**

3. I do not have major comments on the studies conducted given they were meant to be proof-of-concept. However, the results could be presented on the light of recent experimental evidence. For instance, the behavior in the public goods game deserves more attention since the potential gains from cooperation in the large sessions is much more salient. The payoff function used in the paper would produce payoffs that are 10 times larger if subjects were to coordinate on the Pareto efficient outcome. Since the chosen strategies are similar across small and large sessions, subjects in large sessions would have experienced much larger payoffs. In a sense, the failure to coordinate is much more consequential.

- **Response: We now discuss this important point you raise about the stakes faced by large versus small groups in play of the public good game in our experiments.**

4. The paper should discuss the scaling of payoffs more in detail. For instance, in the beauty contest, the scale of payoffs can be held constant regardless of the size of the group. So, one can evaluate the effect of group size directly. However, in public good games, the scale of payoff changes with the group size. So, changing the group size keeping constant the parameters of the payoff function do change the scale of payoffs. In other words, one would like to evaluate the effect of changing the parameters of the payoff function holding constant different group sizes (as in Isaac & Walker, 1988). This kind of methodological concern becomes more prominent as the opportunity to scale up the size of experiment emerges. The authors have an opportunity to take a fresh look at these issues in light of their results.

- **Response: We now discuss the correct procedure for rescaling payoffs in the public good game and note that Isaac and Walker [1988] did such a treatment. The difficulty, as we note, is that such a rescaling involves changes to both the MPCR and the group size, and so it is less clear which change matters. To find out, one needs to do both, of course, but this was not practicable in our design with multiple games played all at once.**

5. I find the section discussing behavior across games distracting. First, analysis across games using very large samples is provided by Rubinstein (QJE 2016) where a typology of players is attempted. Empirical relations are very interesting, but it is not clear what is really new in this section. A more detailed discussion of behavior in the large-scale games would be more informative since those are new and puzzling. In this case, there are clear theoretical benchmarks.

- **Response: Following your suggestion, we have expanded upon our discussion of how our large versus small group games map to the literature. We have moved our discussion of behavior across games to the supplementary material.**

References

R. M. Isaac and J. M. Walker. Group size effects in public goods provision: The voluntary contributions mechanism. *The Quarterly Journal of Economics*, 103(1):179–199, 1988.

R. M. Isaac, J. M. Walker, and A. W. Williams. Group size and the voluntary provision of public goods: Experimental evidence utilizing large groups. *Journal of Public Economics*, 54(1):1–36, 1994.
